# A Snail Galactosed Glycosaminoglycan Inhibits Thrombosis without Affecting Hemostasis via Disrupting FIXa–FVIIIa Complex Generation

**DOI:** 10.1021/acscentsci.5c02230

**Published:** 2026-03-18

**Authors:** Lisha Lin, Debing Pu, Nanyu Xiong, Dong Meng, Zhongtang Li, Xi Gong, Ya Li, Jiangyan Chen, Jiang Li, Lian Yang, Chuang Xiao, Chan Zhang, Xudong Dong, Mingyi Wu

**Affiliations:** † State Key Laboratory of Phytochemistry and Natural Medicines, 26445 Kunming Institute of Botany, Chinese Academy of Sciences, Kunming 650201, China; ‡ School of Traditional Chinese Pharmacy, Shenyang Pharmaceutical University, Shenyang 117004, China; § University of Chinese Academy of Sciences, Beijing 100049, China; ∥ School of Pharmacy, Dali University, Dali 671003, China; ⊥ State Key Laboratory of Natural and Biomimetic Drugs, School of Pharmaceutical Sciences, 33133 Peking University, Beijing, 100191, China; # The First People’s Hospital of Yunnan Province, 74621 The Affiliated Hospital of Kunming University of Science and Technology, Kunming 650032, China; g School of Pharmaceutical Science and Yunnan Key Laboratory of Pharmacology for Natural Products, 71240 Kunming Medical University, Kunming 650500, China

## Abstract

Thrombosis underlies many life-threatening cardio-cerebrovascular diseases. Although existing anticoagulants are effective in treating thrombotic diseases, their application is limited due to the concern of bleeding. New anticoagulants that preserve hemostasis have significant clinical importance. Herein, a novel galactosylated glycosaminoglycan, with unique sequence and sulfate substitutions, was isolated from the snail *Camaena cicatricose* (CCG). Administration of CCG effectively inhibited thrombus formation in a rat venous thrombosis model, which is positively correlated with its *ex vivo* anticoagulant activity (APTT prolongation), with much lower bleeding risk compared with heparins. It is also effective in preventing thrombosis in the rat arterial-venous shunt model and endotoxin-treated mice. CCG inhibited coagulation by selectively targeting iFXase enzyme complex (FIXa–FVIIIa), in an antithrombin (AT)-independent manner. CCG can bind to FIXa with high affinity and decrease the affinity of FIXa–FVIIIa, with no effect on the FIXa activity. Compared with heparins, it cannot bind to AT and exhibits high selectivity for iFXase inhibition, consistent with its absence of the specific heparin pentasaccharide sequence. Overall, the snail galactosed glycosaminoglycan inhibits thrombosis without affecting hemostasis via disrupting iFXase (FIXa–FVIIIa). CCG may represent a promising candidate for thrombosis treatment without increased bleeding risk.

## Graphical abstract

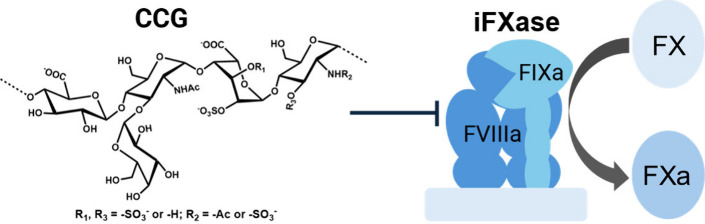

## 1 Introduction

Thrombotic cardiovascular disease is the leading cause of mortality and morbidity globally, being responsible for about 25% of deaths, in which venous thromboembolism (VTE) is the third leading cause after stroke and myocardial infarction.^1,2^ Venous thrombus is commonly formed in the leg vein and results in deep vein thrombosis, or dislodges and causes more life-threatening pulmonary embolism.^3^

Hemostasis, the rapid formation of a clot to seal the injured blood vessel, is essential for limiting blood loss. However, inappropriate activation of the coagulation system and platelets can result in pathological thrombosis. Venous thrombosis primarily arises from blood stasis and coagulation activation.^4^ Increasing studies reveal the interconnection between immunity, inflammation, and the coagulation system, and therefore, infection also contributes to VTE. For instance, sepsis substantially increases VTE risk.^5^ As an essential thrombus component, fibrin is transformed from fibrinogen, catalyzed by thrombin generated from the coagulation cascade. Therefore, anticoagulation is a mainstay therapy for various diseases involving both venous and arterial thrombosis.^6^ However, increased bleeding risk remains the main challenge of approved anticoagulants, such as heparin, warfarin, rivaroxaban, and dabigatran,^7^ for the targeted coagulation factor(s) are also essential for hemostasis.

Out of the great clinical burden, researchers have been seeking safer anticoagulants.^8–10^ Recent evidence indicates that thrombosis and hemostasis mechanisms can be separated under specific pathological conditions, implying the possibility of selective intervention.^11,12^

In the coagulation cascade model, the intrinsic coagulation pathway is triggered by plasma contact activation—factor XII (FXII) is activated when plasma encounters negatively charged macromolecules, such as polyphosphate, cell-free DNA (cfDNA), lipopolysaccharide (LPS), and misfolded protein aggregates, etc., and leads to thrombosis,^13–16^ while the extrinsic coagulation pathway is initiated by the exposure of subendothelial tissue factor (TF) to blood, leading to hemostasis.^17^ Additionally, based on the cell-based coagulation model, a small amount of TF from TF-bearing cells can also induce thrombosis, in which intrinsic coagulation factors play an important role in coagulation amplification and propagation.^18,19^ Consequently, the intrinsic coagulation pathway is involved in pathological thrombosis, and iFXase (FIXa-FVIIIa enzyme complex) assembled in activated platelet membrane is the rate-limiting enzyme.^20,21^ Consistently, epidemiologic and experimental data showed that intrinsic coagulation pathway (FXII, FXI, FIX-FVIII) contributes more to thrombosis than to hemostasis,^22–24^ whereas the extrinsic (FVII-TF) and common (FX-FV, FII) coagulation pathways are indispensable for hemostasis.

Intrinsic coagulation factors become the potential target for developing new-generation anticoagulants.^22^ Such inhibitors exhibit an antithrombotic effect without increasing bleeding in preclinical and clinical studies, including FXII antibody or inhibitor, FXIa small-molecule inhibitor (asundexian and milvexian), and FIXa ribonucleic acid aptamer (pegnivacogin), etc., and some have advanced to Phase II and III clinical trials with encouraging outcomes.^25–29^ Although substantial breakthroughs have been made, more effective inhibitors are required to verify the role of intrinsic coagulation factors and their superiority as anticoagulant targets.

In this work, we discovered a novel glycosaminoglycan with anticoagulant activity from the land snail *Camaena cicatricose* (CCG). We characterized its structure in detail and studied its anticoagulant activity and mechanism and *in vivo* antithrombotic effect. CCG has distinct sulfation substitution and galactosed branches, and shows a unique anticoagulant mechanism by binding to FIXa and antagonizing its binding to FVIIIa, thus selectively inhibiting iFXase in an AT-independent manner. CCG was effective in several animal thrombotic models, with low bleeding risk; as an anticoagulant candidate, it may meet the need for safer long-term anticoagulation.

## 2 Results

### 2.1 A Novel Glycosaminoglycan with Unique Branches Discovered from Snail

A glycosaminoglycan (CCG) was isolated and purified from the gastropod of snail *C. cicatricose*, with the yield of 1.06% (Figure 1A). The HPGPC symmetric singlet indicated that it is a homogeneous polymer (Figure S1A). Additionally, no absorption peak in 260–280 nm was observed in the UV profile (Figure S1B), indicating the absence of proteins and nucleic acids. Its average molecular weight (*M*_w_) was 32.6 kDa (Table S1). Its molar ratio of sulfate to carboxyl group was 1:1.29, as determined by conductometric titration (Figure S1C).

**Figure 1:**
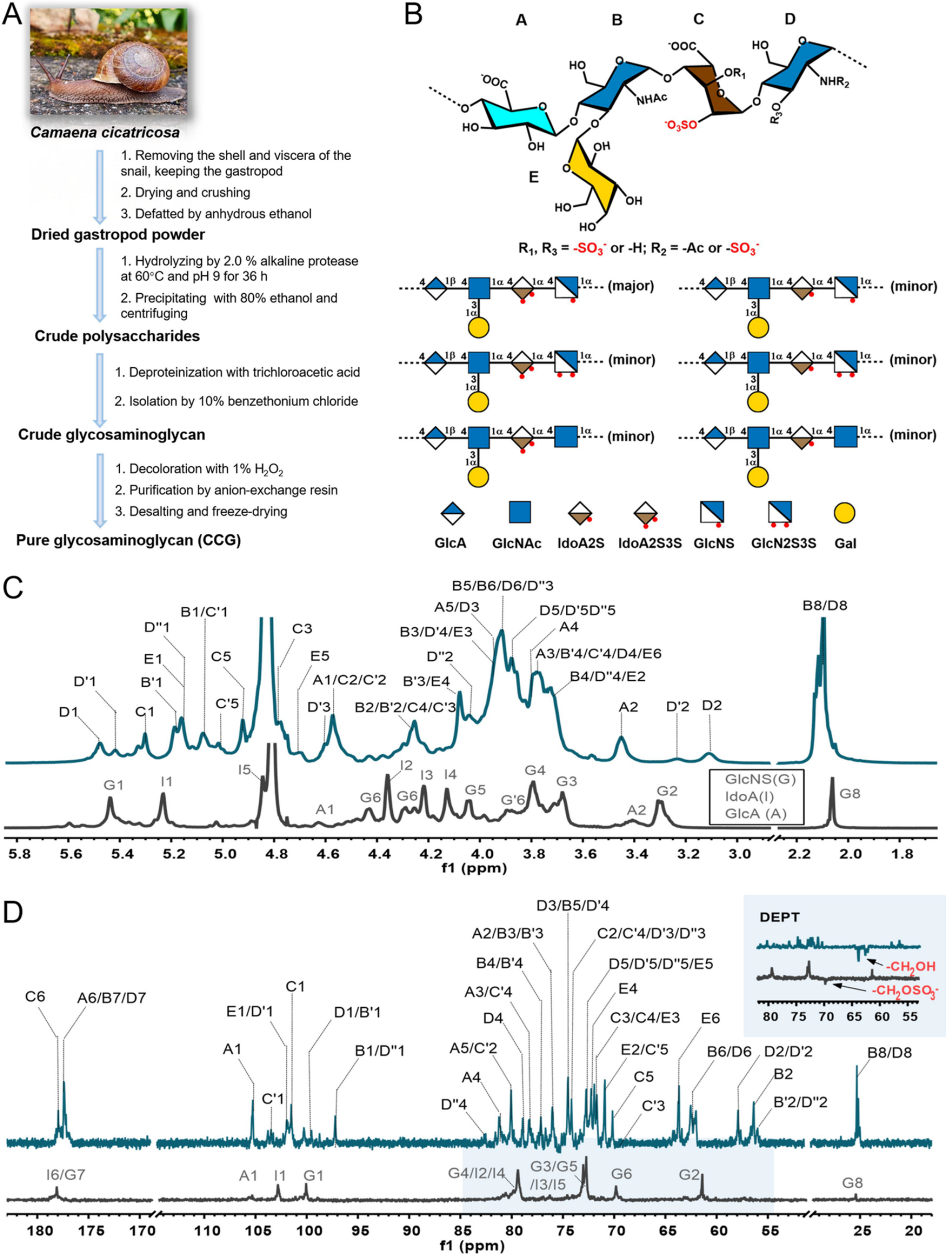
Isolation, chemical structure, and NMR spectra of CCG. (A) Flowchart of isolation and purification of CCG from snails. (B) Proposed chemical structure of CCG. (C) ^1^H NMR spectra of CCG and heparin. (D) ^13^C NMR and partial DEPT spectra of CCG (cyan) and heparin (black). The numbers indicated the positions of ^1^H or ^13^C in monosaccharide residues.

PMP-derivatization analysis revealed that CCG contained four monosaccharide residues: glucuronic acid (GlcA), *N*-acetylglucosamine (GlcNAc), iduronic acid (IdoA), and galactose (Gal) (Figure S1D), implying a new glycosaminoglycan.^30^ During mild acid hydrolysis, the galactoses in CCG were progressively released (Figures S2–S3), and the main chain (CCG-MC) was concurrently produced (Figures S4–S5). Given that polysaccharide side chains are more susceptible to acid hydrolysis, galactoses should be the side chains. In addition, methylation analysis indicated that CCG mainly contains the following monosaccharide residue linkages: terminal-linked-Gal, 1,4-linked-GlcNAc, 1,4-linked-GlcA and/or 1,4-linked-IdoA, and 1,3,4-linked-GlcNAc (Figures S6–S7), and the galactose branches linked to C-3 of GlcNAc.

Although CCG contains a heparin-like backbone, it is markedly different from heparin.^31,32^ Primarily, CCG possesses unique Gal side chains. Moreover, the acetyl (δ_H‑1_ 2.07 ppm) content in CCG is approximately 10-fold higher than that in heparin (Figure 1C). In the DEPT spectra (Figure 1D), according to the methylene signals of heparin (δ_C‑1_ 69 ppm) and CCG (δ_C‑1_ 63 ppm), most of the C-6 of glucosamine in heparin are sulfated but not in CCG. In addition, the ^1^H NMR signal for IdoA H-3 in CCG (δ_H_ 4.75 ppm) appeared at a lower field compared with that in heparin (δ 4.22 ppm), suggesting that its C-3 was sulfated. Overall, CCG has a lower sulfate content than heparin (13.5% and about 25%, respectively). Taken together, CCG embodies a novel glycosaminoglycan with Gal branches.

A degraded CCG (dCCG) was prepared by free radical depolymerization (Figure S8, Table S1), whose NMR profile resembled that of CCG, but with better resolution (Figure S9), indicating that the main structural features remained after depolymerization. Thus, the structure of CCG was elucidated using the NMR spectra of dCCG. According to the 1D and 2D NMR spectra (Figures 1C–D, 2A–B, Figure S10), eight possible monosaccharide residues in dCCG were assigned: GlcA (A), GlcNAc (B), IdoA2S3S (C), IdoA2S (C’), GlcNS (D), GlcN2S3S (D’), GlcNAc (D’’), and terminal-linked Gal (E) (Figure 2E, Tables S2–S3). The linkage order between these residues was mainly assigned by the key HMBC and ROESY cross-peaks (Figure 2C–E). Deduced from the HMBC correlations of B_H‑4_/A_C‑1_, C_H‑4_/B_C‑1_, C_H‑1_/D_C‑4_, A_H‑4_/D_C‑1_, and E_H‑1_/B_C‑3_, the linkage sequence between these residues is →4)-A(1→4)-[E(1→3)]-B(1→4)-C(1→4)-D­(1→ (Table S4). The ROESY correlations of H_B‑4_/H_A‑1_, H_B‑1_/H_C‑4_, H_C‑1_/H_D‑4_, H_D‑1_/H_A‑4_, and H_B‑3_/H_E‑1_, further verified the above linkage type. CCG was composed of a pentasaccharide repeating unit including six possible sequences. Among them, the first pentasaccharide unit is likely the predominating unit (Figure 1B), for the relatively high proportion of residues C and D. Thus, the polysaccharide parent was determined as {→4)-d-GlcA-β­(1→4)-[d-Gal-α­(1→3)]-d-GlcNAc-α­(1→4)-l-IdoA2S­(or 2S3S)-α(1→4)-d-GlcN­(2S, or 2S3S, or 2Ac)-α(1→}­n. The detailed structural elucidation was described in Supporting Information. Overall, CCG was a structurally complex glycosaminoglycan with distinctive Gal branches, sulfation pattern, and sequence (Figure 1B).

**Figure 2:**
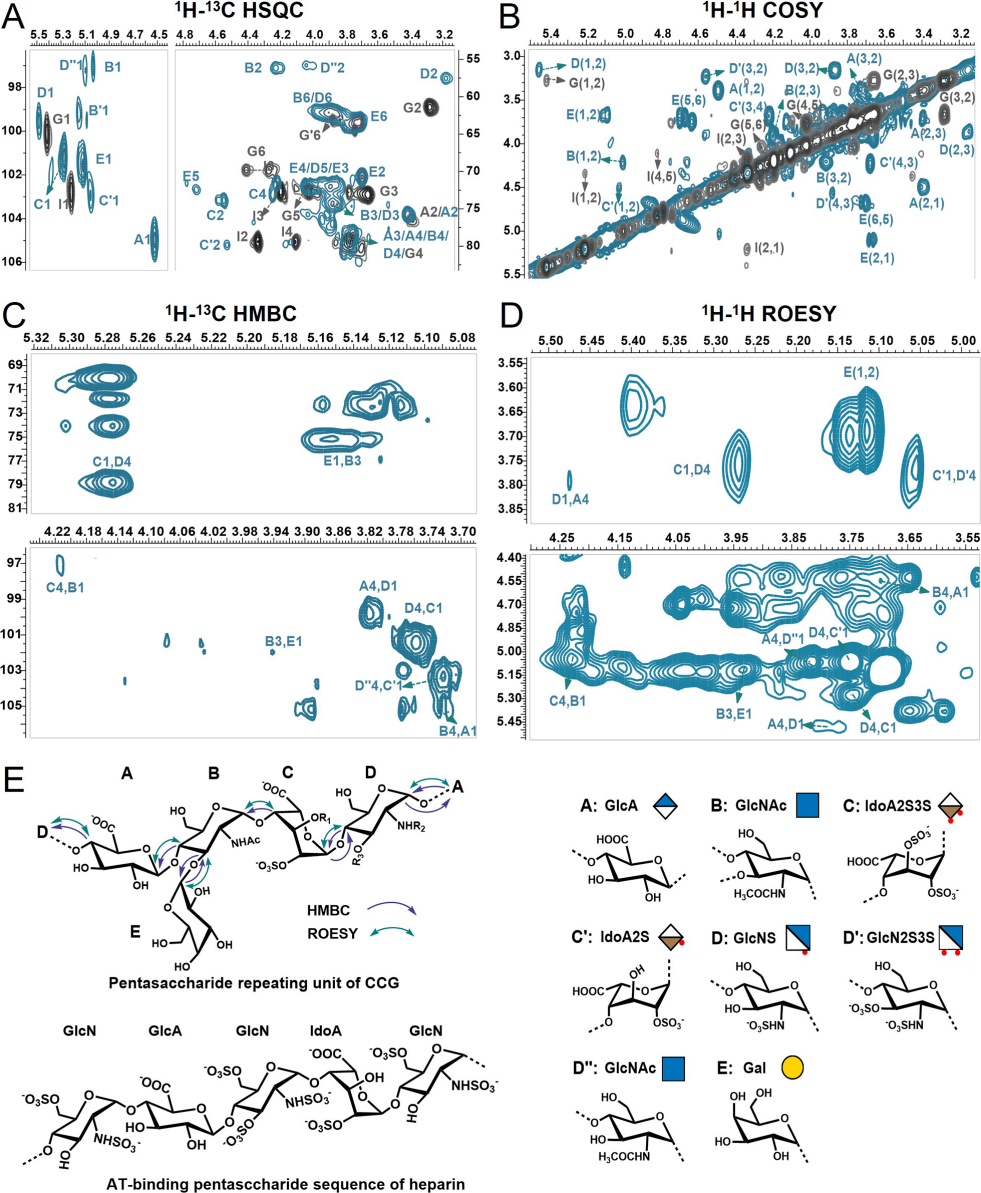
Comparative analysis of the NMR spectra of dCCG and heparin. (A) The overlapped ^1^H–^13^C HSQC spectra of dCCG (cyan) and heparin (gray). (B) The overlapped ^1^H–^1^H COSY spectra of dCCG (cyan) and heparin (gray). (C) The key ^1^H–^13^C correlation in HMBC spectra of dCCG. (D) The key ^1^H–^1^H correlation in ROESY spectra of dCCG. (E) Schematic diagram of HMBC and ROESY correlations, and the structure of pentasaccharide repeating unit of CCG and AT-binding pentasaccharide sequence of heparin, and their monosaccharide residues.

### 2.2 CCG Inhibited Thrombosis by Inhibiting Intrinsic Coagulation Pathway without Increased Bleeding

Given that CCG contains a heparin-like backbone, its anticoagulant activity was studied, compared with CCG-MC and two other snail GAGs, AFG and HLG (from *Achatina fulica* and *Helix lucorum*, respectively). CCG prolonged the activated partial thromboplastin time (APTT) of human plasma, with APTT-doubling concentration (EC_2.0×_) of 95.7 μg/mL, indicating intrinsic coagulation pathway inhibition. And differing from heparins, it had no obvious effect on prothrombin time (PT) and thrombin time (TT) (Figure S11A, Table S5). Compared with CCG, CCG-MC, AFG, and HLG had no obvious anticoagulant activity, indicating that the Gal branches and special sulfate substitution were important.

The antithrombotic activity of CCG was first evaluated in the whole blood system using a thromboelastogram (TEG). CCG at 50 μg/mL significantly prolonged the reaction time (*R*) and decreased the fibrin cross-linking rate (α angle) and maximum amplitude (MA), preliminarily indicating the antithrombotic effect of CCG (Figure S11B).

The *in vivo* antithrombotic effect of CCG was studied in a rat deep venous thrombosis (DVT) model. CCG at 5–20 mg/kg dose-dependently and significantly inhibited thrombus formation, based on the data of thrombus weight and length (Figure 3A–C, Table S6). Its inhibition ratio was about 30% at 5 mg/kg (thrombus dry weight of 11.8 ± 2.2 mg *vs* 18.0 ± 2.1 mg, *P* < 0.001), reached about 70% at 10 mg/kg (thrombus dry weight, 5.5 ± 1.7 mg, *P* < 0.001 *vs* control), and exhibited almost complete inhibition at 20 mg/kg (thrombus dry weight, 0.3 ± 0.2 mg, *P* < 0.001 *vs* control). Consistent with the *in vitro* data, after administration CCG selectively prolonged rat plasma APTT, while heparins also prolonged PT and TT (Figure 3D, Figure S12A–C). Moreover, the antithrombotic activity of CCG was linearly correlated with its APTT prolonging effect (*R* ^2^ > 0.98) (Figure 3E), indicating that CCG exhibits antithrombotic activity by inhibiting the intrinsic coagulation pathway.

**Figure 3:**
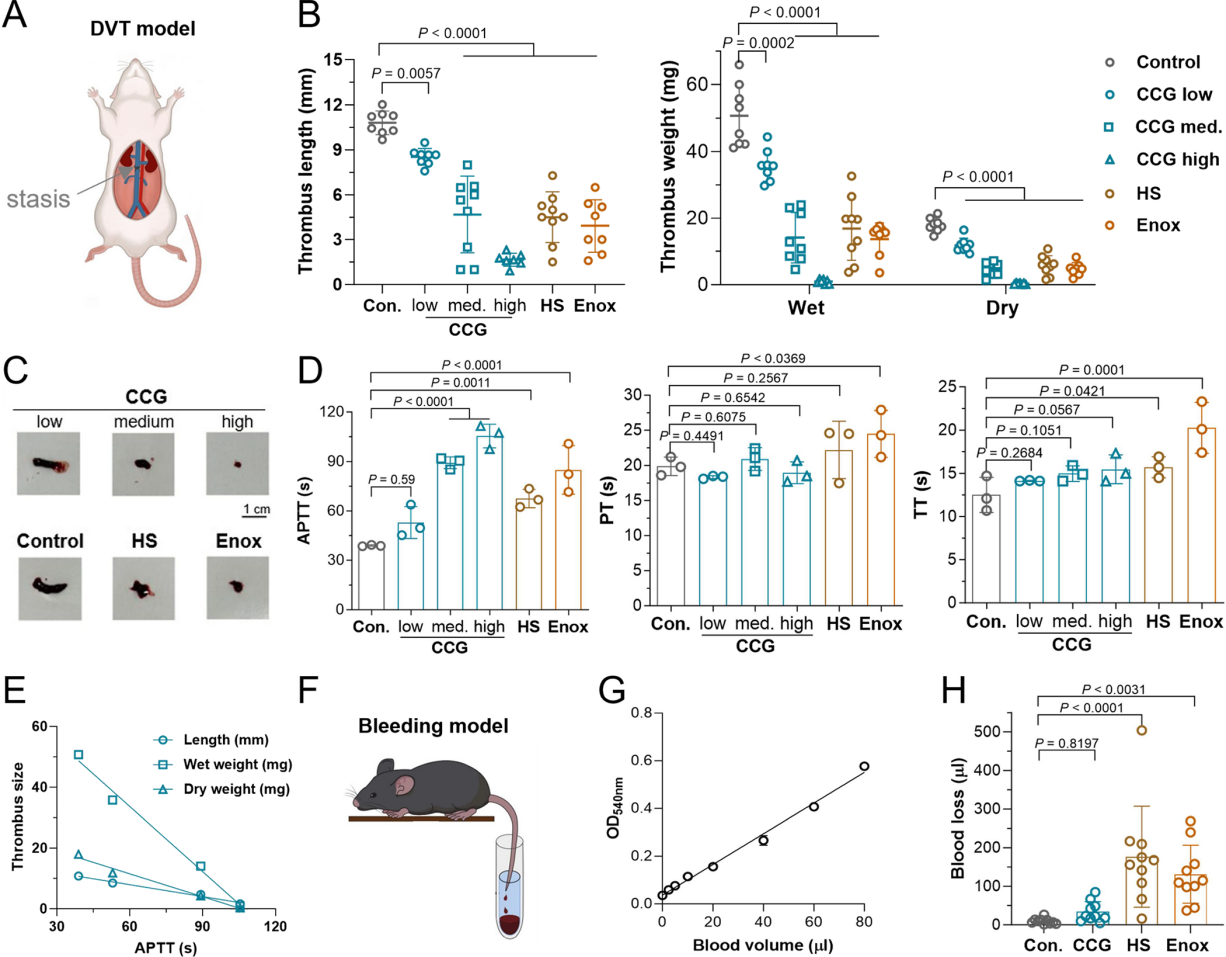
The effects of CCG on venous thrombosis and bleeding. (A) Schematic of the rat DVT thrombosis model. (B) The effect of CCG on thrombus formation, including thrombus length, wet weight, and dry weight, CCG was at 5, 10, and 20 mg/kg, HS was at 1.25 mg/kg, and Enox was at 0.8 mg/kg (mean ± SD, *n* = 7–9). (C) The representative thrombus pictures. (D) The effect of CCG on the coagulation function of rat plasma (mean ± SD, *n* = 3). (E) The correlation between rat plasma APTT and thrombus length, wet weight, or dry weight. (F) Schematic of mouse tail-cut bleeding model. (G) The blood volume-optical density standard curve. (H) The effect of CCG on mouse tail bleeding, CCG was at 70 mg/kg, HS was at 8.75 mg/kg, and Enox was at 5.6 mg/kg (mean ± SD, *n* = 10).

Based on the above results, bleeding risk was evaluated using a mouse tail-cut bleeding model with overdosed (5-fold of antithrombotic dose in rat was transformed to a mouse dose) compound. At the same fold of equivalent antithrombotic dose, CCG did not increase bleeding compared with the control, while HS, Enox, and Hep significantly increased blood loss (Figure 3F–H, Figure S12D). The data indicate that compared with heparins, CCG possessed a lower bleeding risk, consistent with its anticoagulant characteristics, inhibiting the intrinsic but not extrinsic or common coagulation pathway.

### 2.3 CCG Prevented Thrombosis in A-V Shunt and Endoxemia-Enhanced DVT Models

Next, the antithrombotic effect of CCG was further evaluated by a rat arteriovenous (A-V) shunt model, in which a “mixed thrombus” is formed on an exogenous thread (Figure 4A). CCG at 10 mg/kg significantly inhibited thrombus formation (thrombus dry weight, 8.6 ± 2.2 mg *vs* 11.5 ± 2.1 mg, *P* < 0.05), with an inhibition rate of about 25% (Figure 4B, Table S7). In this model, CCG also significantly inhibited rat plasma coagulation (APTT prolongation) (Figure 4C), but compared with the DVT model, CCG was less effective in the A-V shunt model.

**Figure 4:**
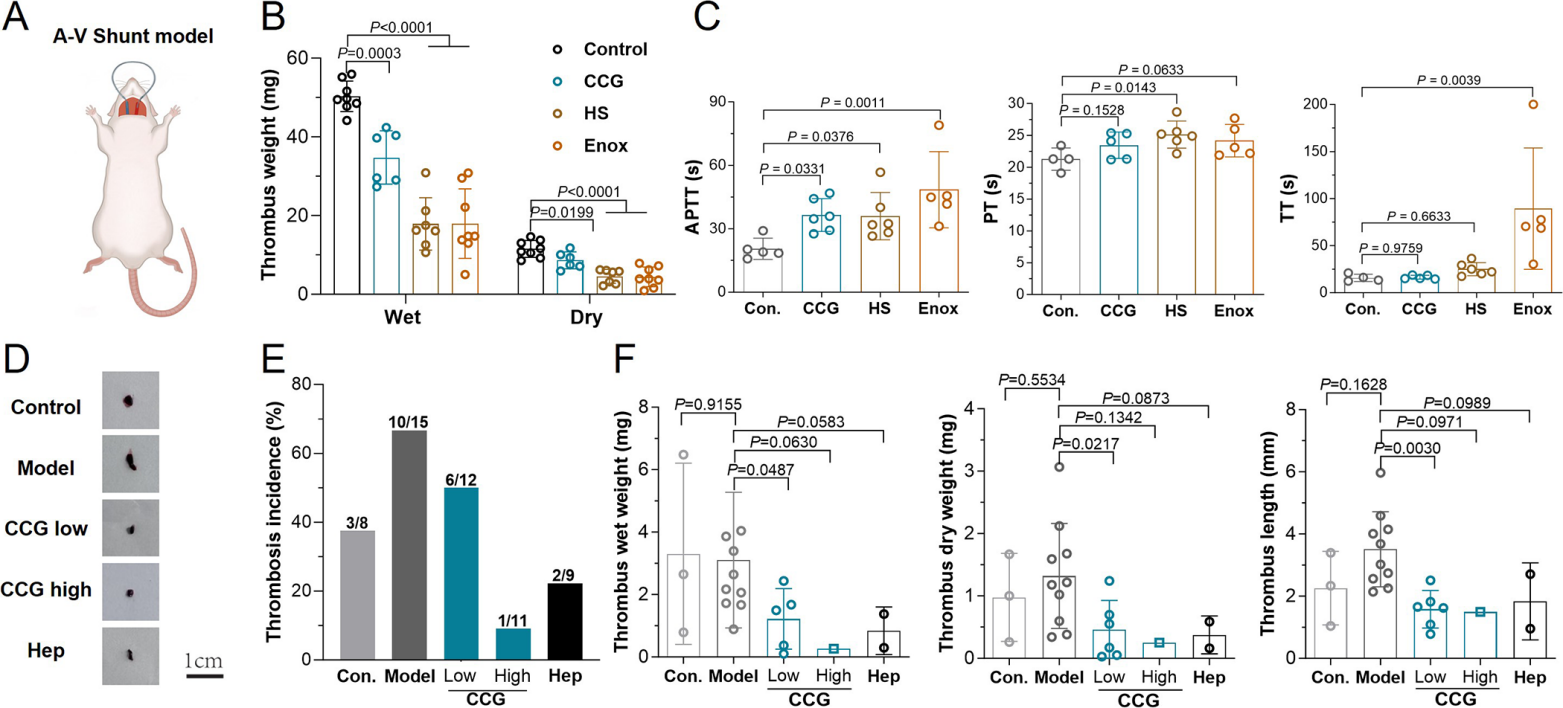
The protection effects of CCG on A-V shunt thrombosis and endotoxemia-enhanced DVT models. (A) Schematic of rat A-V shunt thrombosis model. (B) The effect of CCG on thrombosis in rat A-V shunt model, CCG was at 10 mg/kg, HS and Enox were at 5 mg/kg (mean ± SD, *n* = 6–8). (C) The effect of CCG on the coagulation function of rat plasma from the rat A-V shunt model (mean ± SD, *n* = 4–6). (D) The representative thrombus pictures from mouse LPS-treated DVT model. (E–F) The thrombosis incidence (E) and thrombus weight and length (F) in a mouse LPS-treated DVT model (mean ± SD). For thrombus wight and length, only those >0 mg were included. CCG was at 5 and 10 mg/kg, Hep was at 1 mg/kg.

Immunothrombosis is recognized as a convergent result of immunity, inflammation, and coagulation, in which the intrinsic coagulation pathway contributes to thrombus propagation.^18^ The pathogen molecule endotoxin or LPS can trigger coagulation by activating the plasma contact system and inducing TF expression in monocytes. Thus, sepsis substantially increases the risk of venous thrombosis.^15,33^ In a mouse DVT model, after the inferior vena cava (IVC) was ligated for 6 h without inducer, the thrombosis incidence was 37.5% (3/8), which was almost doubled with LPS treatment (66.7%, 10/15), although thrombus were not significantly increased by LPS (thrombus wet weight, 3.1 ± 2.2 mg *vs* 3.3 ± 2.9 mg, *P* = 0.9155) (Figure 4D–F, Table S8). CCG can effectively reduce thrombosis incidence (50% for 5 mg/kg group, and 9.1% for 10 mg/kg group), thrombus length, and weight. Compared with heparin (1 mg/kg), CCG at 10 mg/kg showed a superior effect, with a lower bleeding risk (Figure 4H, Figure S12D). Additionally, after LPS treatment, the inflammation factors IL-6 and TNF-α were largely increased, on which CCG had no significant effect (Figure S13). It indicated that CCG inhibits endotoxin-associated thrombosis mainly by coagulation regulation.

### 2.4 CCG Inhibited iFXase Activity by Disrupting FIXa–FVIIIa Complex Generation

The classic drug heparins exhibit anticoagulant activity by facilitating an inactivation effect of AT against several coagulation factors, including FXa and FIIa in a common pathway, and FIXa and FXIa in the intrinsic pathway.^17,34^ Since CCG does not contain the AT-binding heparin pentasaccharide, its anticoagulant mechanism may be different. Indeed, in the presence of AT, CCG had no obvious effect on FXa and FIIa (IC_50_, >5000 ng/mL) (Figure 5A–B), whereas CCG potently inhibited iFXase activity (IC_50_, 25.8 ng/mL) independent of AT (Figure 5C, Table S5). By contrast, heparin and enoxaparin can inhibit the FXa, FIIa, and iFXase indistinguishably, IC_50_ values were all less than 100 ng/mL, HS also moderately inhibited FXa and FIIa in the presence of AT, with much weaker activity against iFXase (Figure 5A–C). FXa and FIIa in the common coagulation pathway are essential for hemostasis, and the fact that CCG had no effect on these two coagulation factors is consistent with its lower bleeding risk (Figure 4H).

**Figure 5:**
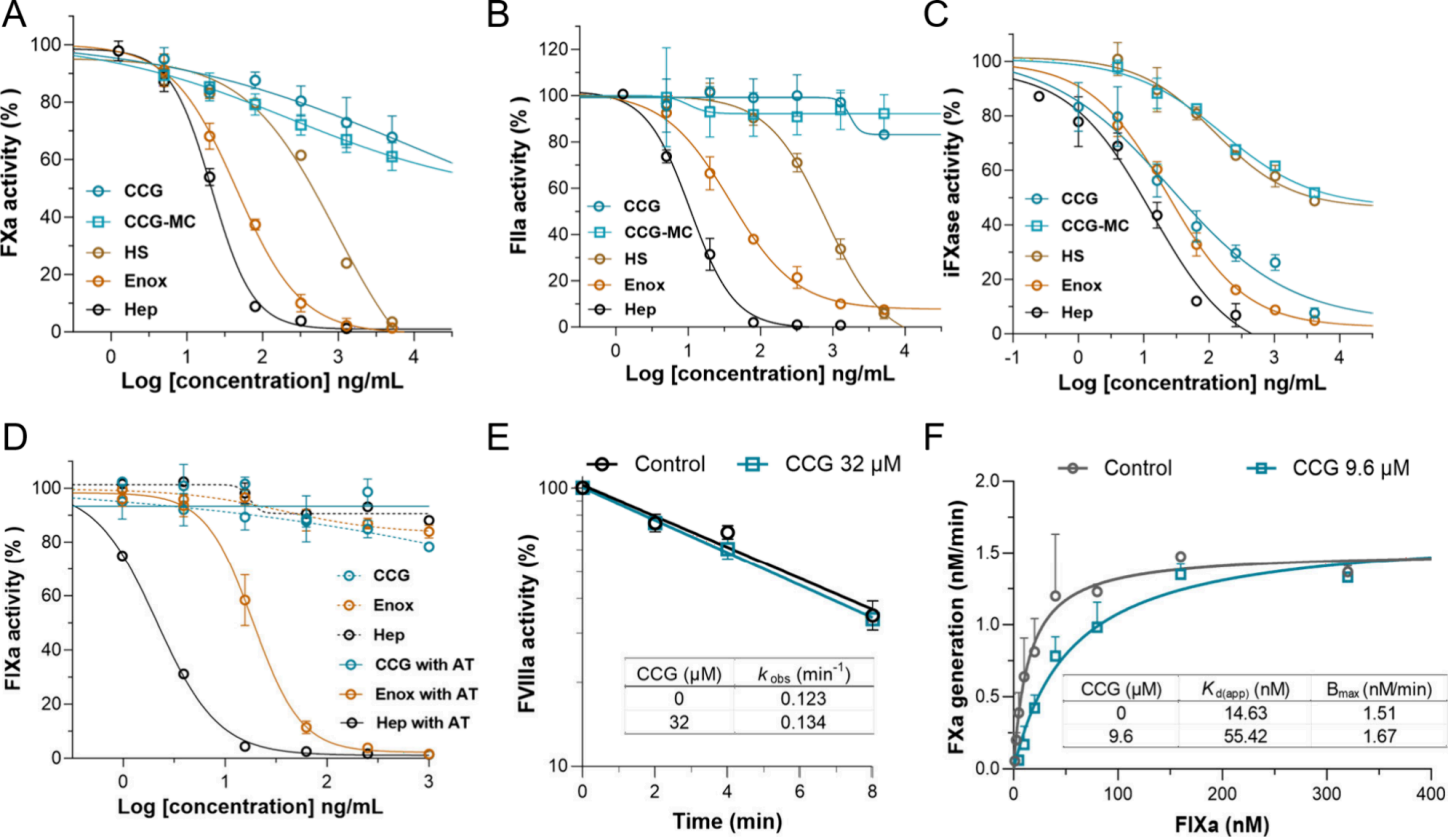
CCG inhibited iFXase complex by disrupting FIXa and FVIIIa binding. (A–D) The effect of snail GAGs on the activity of FXa (A), FIIa (B), iFXase (C), and FIXa (D). (E) The effect of CCG on the decay rate of FVIIIa activity. (F) The effect of CCG on the apparent affinity of FIXa and FVIIIa. Data were presented as mean ± SD; experiments were conducted in triplicate or duplicate.

The iFXase is the last and rate-limiting enzyme in the intrinsic coagulation pathway, composed of FIXa and cofactor FVIIIa; the assembly of FIXa and FVIIIa also requires phospholipid and Ca^2+^. However, CCG had no effect on the activity of FIXa, with or without AT (Figure 5D). When circulating in blood, FVIIIa is stable by forming a noncovalent complex with von Willebrand factor (vWF). Once activated by thrombin, FVIIIa dissociates from vWF and interacts with FIXa. FVIIIa is unstable, and the activity degrades in a first-order manner. The effect of CCG on FVIIIa stability was studied, and the observed first-order rate constant (*k*_obs_) was 0.123 min^–1^ without CCG and 0.134 min^–1^ in the presence of CCG, indicating a slight effect of CCG (Figure 5E). Next, the effects of CCG on the affinity of FIXa and FVIIIa were investigated by monitoring iFXase activity at a limiting concentration of FVIIIa and a series concentration of FIXa.^35^ The apparent affinity constant (*K*_D(app)_) of FIXa and FVIIIa was largely increased from 14.6 to 55.4 nM in the presence of CCG, indicating that CCG reduced their binding (Figure 5F).

### 2.5 CCG Selectively Targeted iFXase by Binding to FIXa

Since CCG decreased the *K*_D(app)_ of FIXa and FVIIIa, the interaction of CCG with each of them was further analyzed. Biolayer interferometry (BLI) analysis showed that CCG could bind to FIXa but not FVIIIa at the same concentration (Figure S14). Further study showed that CCG bound to FIXa with high affinity (*K*_D_, 4.6 × 10^–8^ M), while no AT-binding signal was detected (Figure 6A–B, Table S9). Consequently, CCG selectively inhibited the iFXase activity, while it had no AT-dependent inhibitory effect on other coagulation factors, including FIIa, FXa, FIXa, FXIa, and FXIIa (Figure 6C, Table S5). By contrast, heparin can bind to both FIXa and AT, with a *K*_D_ value of 1.8 × 10^–8^ M and 8.8 × 10^–8^ M, respectively (Figure 6D–E, Table S9), which can explain the nonselective effects of heparin and enoxaparin, including AT-independent anti-iFXase, and AT-dependent anti-FXa, anti-FIIa, anti-FIXa, and anti-FXIa (Figure 6F, Figure S15, Table S5).

**Figure 6:**
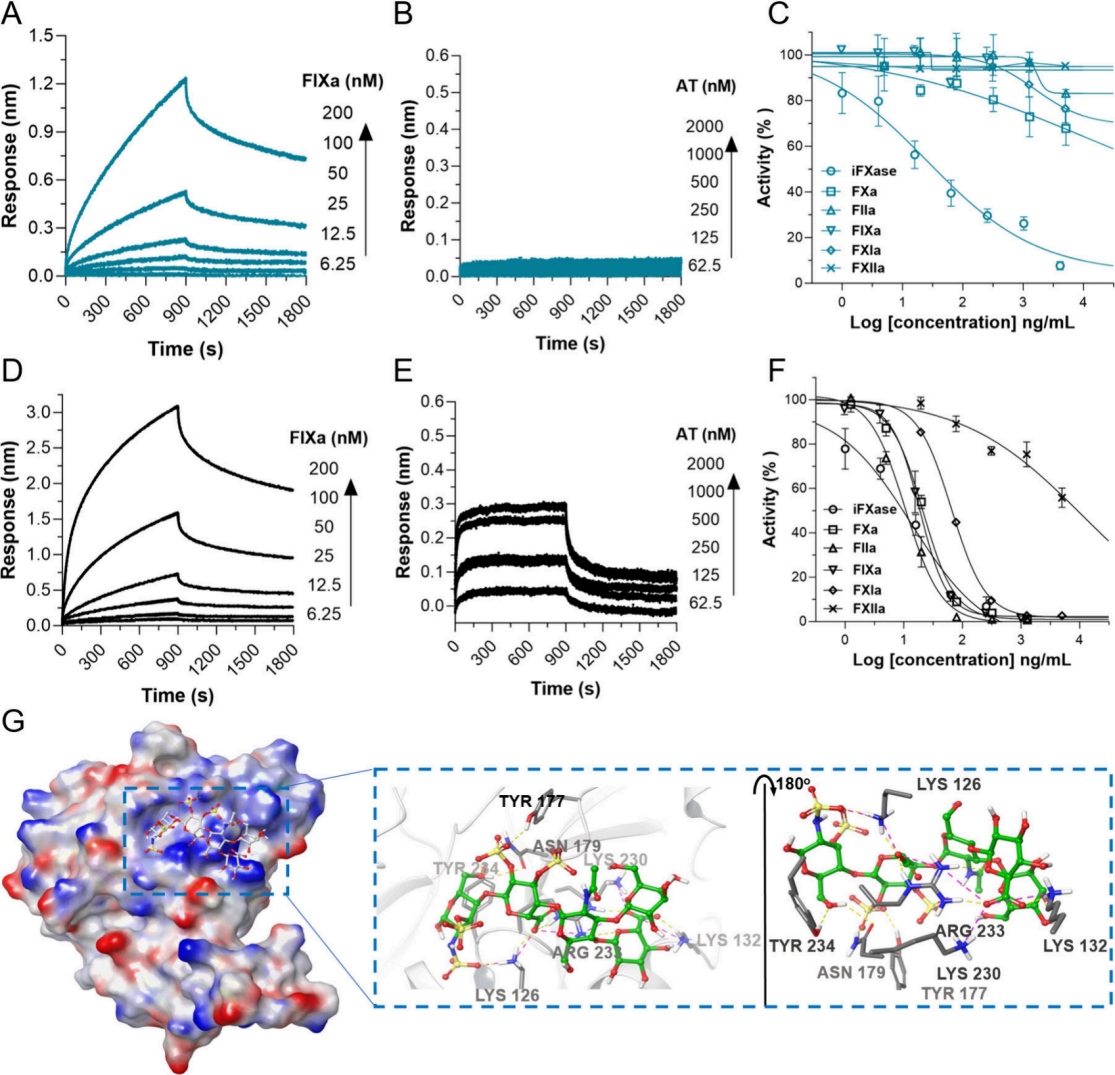
The selectivity of CCG against iFXase. (A–B) The interaction kinetics between CCG and FIXa (A) or AT (B). (C) The effect of CCG on the activity of different coagulation factors. (D–E) The interaction kinetics between heparin and FIXa (D) or AT (E). (F) The effect of heparin on the activity of different coagulation factors. (G) The binding domain of FIXa to CCG. The effect of compounds on iFXase was detected without AT, and their effects on FXa, FIIa, FIXa, FXIa, and FXIIa were detected in the presence of AT. Data are presented as mean ± SD; experiments were conducted in triplicate or duplicate.

Subsequently, the binding site of CCG was studied by a modeling study, referring to the cocrystal structure of the DTRI-177–FIXa complex (PDB ID: 8EPK).^36^ The deep groove between the Lys132-helice and Arg233-helice was selected to fit the oligosaccharide. The CCG pentasaccharide unit bounds to the exosite in the catalytic domain with a high docking score (−8.42), and mainly interacted with some basic residues including Lys126, Lys132, Lys230, and Arg233 (Figure 6G). Its carboxyl and sulfate groups can form hydrogen bonds and salt bridges with the basic residues of FIXa.

As a negatively charged macromolecule, sulfated polysaccharide may induce plasma contact activation, which can be procoagulant via the FXIIa-FXIa pathway and proinflammatory via the kininase-bradykinin pathway, and it may also induce platelet aggregation and thrombopenia.^37–39^ In 2008, the heparin contaminant oversulfated chondroitin sulfate (OSCS) induced severe side effects in patients via plasma contact activation.^40^ OSCS at 1.5–48 μg/mL potently enhanced plasma contact activation, while CCG up to 48 μg/mL showed no obvious effects. In terms of this side effect, CCG was safer than enoxaparin and heparin, with as low activity as HS (Figure S16A). Additionally, CCG had no obvious effect on platelet aggregation (Figure S16B).

Taken together, these data revealed that CCG selectively and AT-independently targeted iFXase by binding to FIXa and interrupting the FXIa–FVIIIa interaction, with no inhibitory activity against FIXa *per se* and other coagulation factors. The iFXase inhibitory activity of CCG endows its anticoagulant and antithrombotic effects, with low bleeding risk (Figure 7).

**Figure 7:**
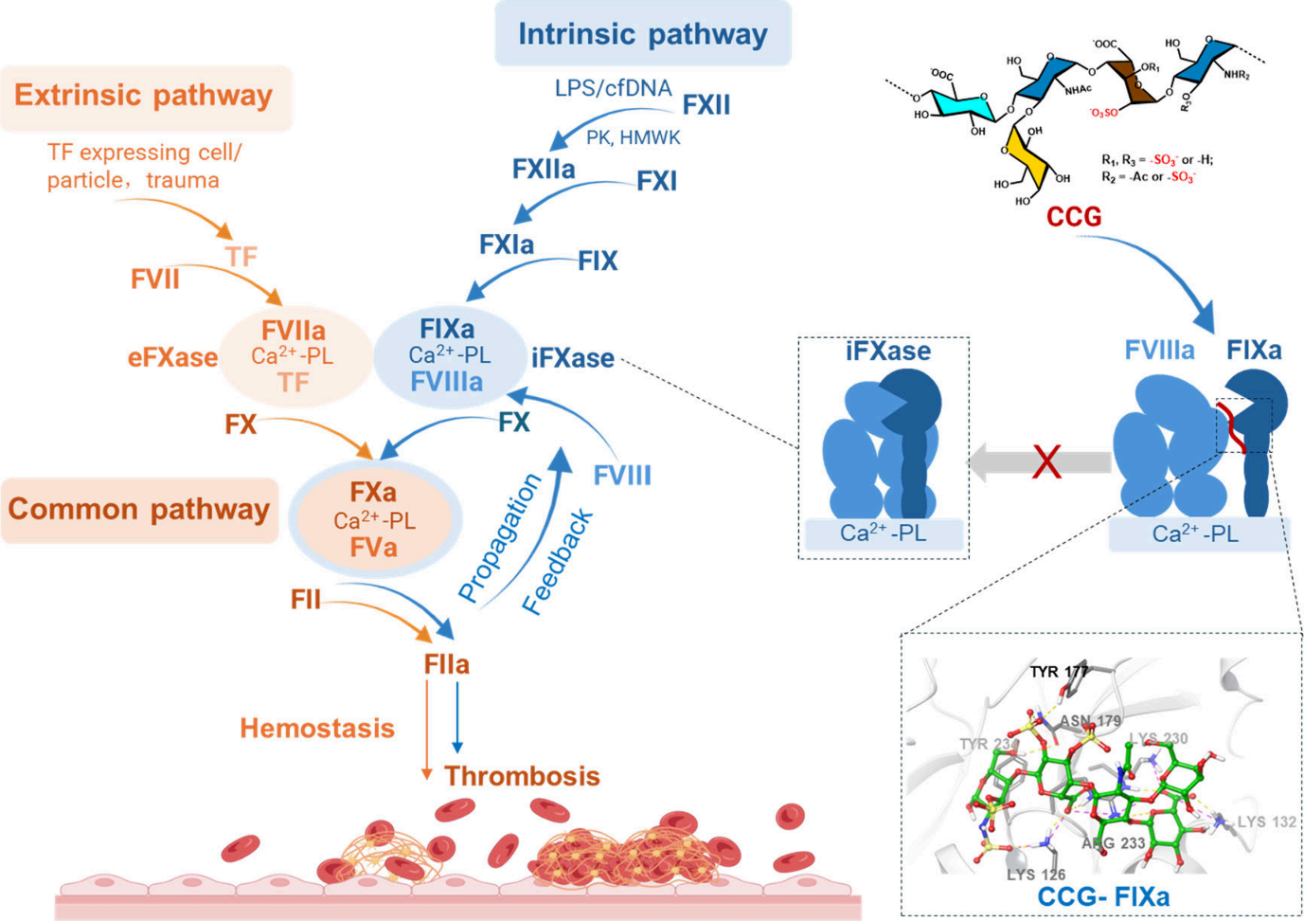
Schematic interpretation of the mechanism and activity of CCG in thrombotic disease.

## 3 Discussion

Anticoagulants are widely used in clinics for the prevention and treatment of thrombotic cardiovascular diseases. However, the targeted coagulation factors are also essential for physiological hemostasis, and thus current anticoagulant drugs have the common defect of increased bleeding risk.^7^

Traditional anticoagulants heparin and warfarin are both effective but nonselective. Heparin inhibits FXIa, FIXa, FXa, and FIIa in an AT-dependent manner, and warfarin inactivates FVIIa, FIXa, FXa, and FIIa by inhibiting vitamin K epoxide reductase complex subunit 1 (VKORC1).^17^ Compared with these two classic anticoagulants, direct oral anticoagulants (DOACs) specifically inhibit FXa or FIIa, with more predictable pharmacodynamics. However, due to their impact on common pathway coagulation factors, bleeding tendency remains.^41^

Based on the advance of perception in thrombosis, intrinsic coagulation factor inhibitors, as a new class of low-bleeding-risk antithrombotic, has shown encouraging outcomes, including small molecule inhibitor, antibody, and RNA aptamer.^26,36,42^ Among others, natural products are an important source. For instance, peptides from forest leech (*Haemadipsa sylvestris*) and bat (*Myotis myotis*) protected against thrombosis and ischemic stroke by inhibiting FXIIa and kallikrein,^43,44^ a protein derived from the blood feeding insect *Triatoma infestans* was found to be an FXIIa inhibitor,^45^ and a glycoprotein from the nose-horned viper (*Vipera ammodytes*) exhibited potent anticoagulation by targeting FVIIIa and inhibiting iFXase.^46^

Our group has also been seeking bioactive sulfated glycosaminoglycan (GAG) from different mollusk resources. We found a GAG from the slug *L. flavus*, with the structure of [→4)-l-IdoA2R-α­(1→4)-d-GlcNAc→α­(1→]­n, has the activity of heparanase inhibition.^47^ And a heparin-like GAG [→4)-d-GlcNAc-α­(1→4)-l-IdoA2S-α­(1→]­n from the snail *A. fulica* can promote diabetic wound healing.^48^ Recently, we also purified galactosylated GAG from the snail *H. lucorum* with strong pro-healing activity.^49^ Although structurally they all belong to heparin GAGs, none of them possess anticoagulant activity. Besides, some GAGs from marine mollusks are reported to exhibit anticoagulant activity by a heparin-like mechanism (AT-dependent FXa inhibition), including a fucosylated heparan sulfate and a heparan sulfate from the scallop *Patinopecten yessoensis* and *Chlamys farreri*, respectively.^50,51^

To our knowledge, CCG is the first reported snail polysaccharide that contains unique galactose branches and exhibits anticoagulant activity (Table S10). The novel snail glycosaminoglycan has a distinctive sulfation pattern and unique Gal branches. Its structure was characterized as {→4)-d-GlcA-β­(1→4)-[d-Gal-α­(1→3)]-d-GlcNAc-α­(1→4)-l-IdoAR_1_-α­(1→4)-d-GlcNR_2_-α­(1→}­n, where R_1_ is 2S3S or 2S, R_2_ is 2S, 2S3S, or 2Ac (Figure 1B). CCG potently targeted the iFXase complex (IC_50_, 25.8 ng/mL) by binding to FIXa and disrupting the interaction between FIXa and its cofactor FVIIIa, while it had no effect on the activity of FIXa and FVIIIa *per se* (Figure 5). Accordingly, CCG may not affect other biofunctions of FIXa, such as the weak FX activation activity in the absence of FVIIIa, and the FVII-TF activation activity.^52^ Consisting of the absence of AT-binding heparin pentasaccharide sequence, CCG did not bind to AT and showed high selectivity in inhibiting iFXase (Figure 6). By contrast, heparin indistinguishably inhibits multiple coagulation factors by AT, including FXa and FIIa in the common coagulation pathway, which can explain its strong effect on APTT prolongation, whereas CCG is more similar to fondaparinux, both of them do not inhibit FIIa and show weaker activity on APTT.

The iFXase complex (FIXa–FVIIIa), assembled on the membrane of activated platelets, is the last and rate-limiting enzyme in the intrinsic coagulation pathway; it is responsible for coagulation propagation and plays an important role in thrombosis. Compared with direct inhibitors of proteinase, iFXase inhibitor blocks iFXase activity by interrupting FIXa–FVIIIa interaction, which represents a promising antithrombotic.^36,46,53–55^ Currently, a fucosylated glycosaminoglycan derivative dHG-5 is the first and only iFXase inhibitor approved for clinical trials.^56–58^

In this work, we identified a novel galactosed glycosaminoglycan CCG, which provided effective protection against various thrombosis, with an action mechanism different from the available anticoagulant drugs. By targeting iFXase, CCG showed an effective and safe antithrombotic effect. In a rat DVT model, CCG dose-dependently inhibited venous thrombosis, and the antithrombotic activity was highly correlated with its *ex vivo* anticoagulant activity (Figure 3). Additionally, administration of CCG induced a much lower bleeding risk compared with heparins, when their dosages were set as the same fold of equivalent antithrombotic dose, aligning with the results that CCG had no effect on the bleeding-related PT and TT (Figure 4). The data suggest a wide therapeutic window of CCG, which may offer therapeutic advantages for patients with bleeding contraindication, such as elderly patients and those with renal failure, as well as for safer long-term anticoagulation.^11^ Moreover, immunothrombosis is recognized as a convergent result of immunity, inflammation, and coagulation.^18^ In an endoxemia-enhanced mouse DVT model, CCG effectively prevented venous thrombosis by reducing both thrombosis incidence and thrombus size (Figure 4), indicating its efficacy for infectious thrombosis.

For future applications, the toxicology, pharmacokinetics, and pharmacodynamics of CCG require more study. Additionally, due to the extreme complexity of its structure, a large amount of synthetic work for constructing the oligosaccharide library is required to fully elucidate the structure–activity relationship. Nevertheless, this work offers a promising candidate molecule; given its favorable safety–efficacy profile, CCG holds promise as a next-generation anticoagulant for use in high-risk populations.

## 4 Conclusions

A new polysaccharide CCG with unique galactose branches was first found from the snail *C. cicatricose*. CCG selectively targets iFXase by binding to FIXa and interrupting the FXIa–FVIIIa interaction, and exhibits AT-independent anticoagulant activity. The novel glycosaminoglycan possesses effective antithrombotic activity with a lower bleeding tendency compared with heparins. The discovery of CCG as a novel iFXase inhibitor also further proves that iFXase is a promising antithrombotic target.

## Supplementary Material



## Notes

Materials and methods, Structural characterization of CCG, Supplementary references, Supplementary figures and tables (PDF)

## References

[1] Virani S. S., Alonso A., Benjamin E. J., Bittencourt M. S., Callaway C. W., Carson A. P., Chamberlain A. M., Chang A. R., Cheng S., Delling F. N., Djousse L., Elkind M. S.V., Ferguson J. F., Fornage M., Khan S. S., Kissela B. M., Knutson K. L., Kwan T. W., Lackland D. T., Lewis T. T., Lichtman J. H., Longenecker C. T., Loop M. S., Lutsey P. L., Martin S. S., Matsushita K., Moran A. E., Mussolino M. E., Perak A. M., Rosamond W. D., Roth G. A., Sampson U. K.A., Satou G. M., Schroeder E. B., Shah S. H., Shay C. M., Spartano N. L., Stokes A., Tirschwell D. L., VanWagner L. B., Tsao C. W. (2020). Heart Disease and
Stroke Statistics-2020 Update: A report from the American Heart Association. Circulation.

[2] Heit J. A., Spencer F. A., White R. H. (2016). The epidemiology
of venous thromboembolism. J. Thromb Thrombolysis.

[3] Lutsey P. L., Zakai N. A. (2023). Epidemiology and
prevention of venous thromboembolism. Nat. Rev.
Cardiol.

[4] Turpie A. G., Esmon C. (2011). Venous and arterial thrombosis--pathogenesis and the rationale for
anticoagulation. Thromb Haemost.

[5] Colling M. E., Tourdot B. E., Kanthi Y. (2021). Inflammation,
infection and venous
thromboembolism. Circ. Res..

[6] Chan N. C., Eikelboom J. W., Weitz J. I. (2016). Evolving treatments for arterial
and venous thrombosis: Role of the direct oral anticoagulants. Circ. Res..

[7] Piran S., Schulman S. (2019). Treatment of bleeding complications
in patients on
anticoagulant therapy. Blood.

[8] Plow E. F., Wang Y., Simon D. I. (2018). The search
for new antithrombotic
mechanisms and therapies that may spare hemostasis. Blood.

[9] Chen S.-C., Qin X., Xiong N., Lin L., Wu Y., Li Q., Sun D., Xiong D.-C., Callmann C. E., Wu M., Ye X.-S. (2025). Comprehensive
synthesis and anticoagulant evaluation of a diverse fucoidan library. Nat. Commun..

[10] Yao W., Chen F., Lin L., Gong X., Guan W., Liu C., Wu M., Ye X.-S. (2026). Automated on-demand synthesis of
a comprehensive library of heparin-like Ulvan oligosaccharides for
decoding their structure-anticoagulant activity relationships. CCS Chemistry.

[11] Mackman N., Bergmeier W., Stouffer G. A., Weitz J. I. (2020). Therapeutic strategies
for thrombosis: new targets and approaches. Nat. Rev. Drug Discov.

[12] Cohen O., Santagata D., Ageno W. (2024). Novel horizons in anticoagulation:
the emerging role of factor XI inhibitors across different settings. Haematologica.

[13] Nickel K. F. (2015). The polyphosphate-factor XII pathway drives
coagulation in prostate
cancer-associated thrombosis. Blood.

[14] Medeiros S. K., Zafar N., Liaw P. C., Kim P. Y. (2019). Purification of
silica-free DNA and characterization of its role in coagulation. J. Thromb Haemost.

[15] Lira A. L. (2025). The physicochemical properties of lipopolysaccharide chemotypes regulate
activation of the contact pathway of blood coagulation. J. Biol. Chem..

[16] Zamolodchikov D., Renné T., Strickland S. (2016). The Alzheimer’s disease peptide
beta-amyloid promotes thrombin generation through activation of coagulation
factor XII. J. Thromb Haemost.

[17] Lin L. (2020). From multi-target anticoagulants
to DOACs, and intrinsic coagulation
factor inhibitors. Blood Rev..

[18] Yong J., Toh C.-H. (2023). Rethinking coagulation:
from enzymatic cascade and
cell-based reactions to a convergent model involving innate immune
activation. Blood.

[19] Hasan S. (2018). Factor IX from prothrombin
complex concentrate augments low dose
tissue factor-triggered thrombin generation *in vitro*. Br J. Anaesth.

[20] Swieringa F., Kuijpers M. J., Lamers M. M., van der Meijden P. E., Heemskerk J. W. (2015). Rate-limiting roles of the tenase
complex of factors
VIII and IX in platelet procoagulant activity and formation of platelet-fibrin
thrombi under flow. Haematologica.

[21] Childers K. C. (2022). SAXS analysis of the
intrinsic tenase complex bound to a lipid nanodisc
highlights intermolecular contacts between factors VIIIa/IXa. Blood Adv..

[22] Fredenburgh J. C., Weitz J. I. (2021). New anticoagulants:
Moving beyond the direct oral anticoagulants. J. Thromb Haemost.

[23] Rietveld I. M. (2019). High levels of coagulation factors and venous thrombosis risk: strongest
association for factor VIII and von Willebrand factor. J. Thromb Haemost.

[24] Ivanciu L., Arruda V. R., Camire R. M. (2023). Factor IXa variants resistant to
plasma inhibitors enhance clot formation in vivo. Blood.

[25] Wilbs J., Kong X.-D., Middendorp S. J., Prince R., Cooke A., Demarest C. T., Abdelhafez M. M., Roberts K., Umei N., Gonschorek P., Lamers C., Deyle K., Rieben R., Cook K. E., Angelillo-Scherrer A., Heinis C. (2020). Cyclic peptide FXII
inhibitor provides safe anticoagulation in a thrombosis model and
in artificial lungs. Nat. Commun..

[26] Xu P., Zhang Y., Guo J., Li H., Konrath S., Zhou P., Cai L., Rao H., Chen H., Lin J., Cui Z., Ji B., Wang J., Li N., Liu D.-P., Renne T., Wang M. (2024). A single-domain antibody
targeting factor XII inhibits both thrombosis and inflammation. Nat. Commun..

[27] Heitmeier S. (2022). Pharmacological profile
of asundexian, a novel, orally bioavailable
inhibitor of factor XIa. J. Thromb Haemost.

[28] Wong P. C. (2022). Milvexian, an orally
bioavailable, small-molecule, reversible, direct
inhibitor of factor XIa: In vitro studies and in vivo evaluation in
experimental thrombosis in rabbits. J. Thromb
Haemost.

[29] Lincoff A.
M. (2016). Effect
of the REG1 anticoagulation system versus bivalirudin on outcomes
after percutaneous coronary intervention (REGULATE-PCI): a randomised
clinical trial. Lancet.

[30] Jackson R. L., Busch S. J., Cardin A. D. (1991). Glycosaminoglycans:
molecular properties,
protein interactions, and role in physiological processes. Physiol Rev..

[31] Pomin V. H. (2014). NMR chemical
shifts in structural biology of glycosaminoglycans. Anal. Chem..

[32] Zhang G.-Q. (2017). An efficient anticoagulant candidate: Characterization, synthesis
and *in vivo* study of a fondaparinux analogue Rrt1.17. Eur. J. Med. Chem..

[33] Sachetto A. T. A., Mackman N. (2023). Monocyte tissue factor expression:
lipopolysaccharide
induction and roles in pathological activation of coagulation. Thromb Haemost.

[34] Hemker H. C. (2016). A century
of heparin: past, present and future. J. Thromb
Haemost.

[35] Sheehan J.
P., Walke E. N. (2006). Depolymerized
holothurian glycosaminoglycan and heparin
inhibit the intrinsic tenase complex by a common antithrombin-independent
mechanism. Blood.

[36] Kolyadko V. N., Layzer J. M., Perry K., Sullenger B. A., Krishnaswamy S. (2024). An RNA aptamer exploits exosite-dependent
allostery
to achieve specific inhibition of coagulation factor IXa. Proc. Natl. Acad. Sci. U. S. A..

[37] Lin L., Wu M., Zhao J. (2017). The initiation
and effects of plasma contact activation:
an overview. Int. J. Hematol.

[38] Lin L. (2018). Plasma contact activation
by a fucosylated chondroitin sulfate and
its structure-activity relationship study. Glycobiology.

[39] Lin L. (2021). High-molecular-weight
fucosylated glycosaminoglycan induces human
platelet aggregation depending on alpha­(IIb)­beta(3) and platelet secretion. Platelets.

[40] Kishimoto T. K. (2008). Contaminated heparin associated with adverse
clinical events and
activation of the contact system. N Engl J.
Med..

[41] Kalathottukaren M. T., Haynes C. A., Kizhakkedathu J. N. (2018). Approaches to prevent bleeding associated
with anticoagulants: current status and recent developments. Drug Deliv Transl Res..

[42] Wichaiyo S., Parichatikanond W., Visansirikul S., Saengklub N., Rattanavipanon W. (2023). Determination of the potential clinical
benefits of
small molecule factor XIa inhibitors in arterial thrombosis. ACS Pharmacol Transl Sci..

[43] Zhang Z., Shen C., Fang M., Han Y., Long C., Liu W., Yang M., Liu M., Zhang D., Cao Q., Chen X., Fang Y., Lu Q., Hou Z., Li Y., Liu Z., Lei X., Ni H., Lai R. (2022). Novel contact-kinin
inhibitor sylvestin targets thromboinflammation and ameliorates ischemic
stroke. Cell. Mol. Life Sci..

[44] Cha L., Yang J., Gao J., Lu X., Chang X., Caroline Thuku R., Liu Q., Lu Q., Li D., Lai R., fang m. (2024). Bat-derived oligopeptide LE6 inhibits the contact-kinin
pathway and harbors anti-thromboinflammation and stroke potential. Zool Res..

[45] May F. (2016). FXIIa inhibitor rHA-Infestin-4:
Safe thromboprotection in experimental
venous, arterial and foreign surface-induced thrombosis. Br. J. Hamaetol..

[46] Latinovic Z. (2018). The first intrinsic tenase complex inhibitor with serine protease
structure offers a new perspective in anticoagulant therapy. Thromb Haemost.

[47] He Z., Zhou L., Lin L., Yin R., Zhao J. (2019). Structure
and heparanase inhibitory activity of a new glycosaminoglycan from
the slug *Limacus flavus*. Carbohydr.
Polym..

[48] Wu Y. (2020). A non-anticoagulant
heparin-like snail glycosaminoglycan promotes
healing of diabetic wound. Carbohydr. Polym..

[49] Li Y. (2025). Structural analysis
and accelerating wound healing function of a
novel galactosylated glycosaminoglycan from the snail *Helix
lucorum*. Carbohydr. Polym..

[50] Onishi S. (2023). Fucosylated heparan
sulfate from the midgut gland of *Patinopecten
yessoensis*. Carbohydr. Polym..

[51] Qin Y. (2024). Unique structural characteristics
and biological activities of heparan
sulfate isolated from the mantle of the scallop *Chlamys farreri*. Carbohydr. Polym..

[52] Ndonwi M., Broze G., Bajaj S. P. (2005). The first epidermal
growth factor-like
domains of factor Xa and factor IXa are important for the activation
of the factor VII–tissue factor complex. J. Thromb Haemost.

[53] Buyue Y., Sheehan J. P. (2009). Fucosylated chondroitin
sulfate inhibits plasma thrombin
generation via targeting of the factor IXa heparin-binding exosite. Blood.

[54] Zhao L. (2015). Discovery of an intrinsic tenase complex inhibitor:
Pure nonasaccharide
from fucosylated glycosaminoglycan. Proc. Natl.
Acad. Sci. U. S. A..

[55] Reed C. R. (2022). Aptamer-based factor IXa inhibition preserves hemostasis and prevents
thrombosis in a piglet model of ECMO. Mol. Ther
Nucleic Acids.

[56] Zhou L., Gao N., Sun H., Xiao C., Yang L., Lin L., Yin R., Li Z., Zhang H., Ji X., Zhao J. (2020). Effects of
native fucosylated glycosaminoglycan, its depolymerized derivatives
on intrinsic factor Xase, coagulation, thrombosis, and hemorrhagic
risk. Thromb Haemost.

[57] Wu M. (2015). Anticoagulant and antithrombotic
evaluation of native fucosylated
chondroitin sulfates and their derivatives as selective inhibitors
of intrinsic factor Xase. Eur. J. Med. Chem..

[58] Li H. (2021). Low-molecular-weight
fucosylated glycosaminoglycan and its oligosaccharides
from sea cucumber as novel anticoagulants: A review. Carbohydr. Polym..

