# Supplementary material for: A Snail Galactosed Glycosaminoglycan Inhibits Thrombosis without Affecting Hemostasis via Disrupting FIXa–FVIIIa Complex Generation
Source: ACS Cent Sci. 2026 Mar 18;12(4):446–56. doi: 10.1021/acscentsci.5c02230 (PMC13107218; doi:10.1021/acscentsci.5c02230)
Supplement: Supplementary file 1 [file oc5c02230_si_001.pdf]

Supporting Information for

ORIGINAL ARTICLE

A snail galactosed glycosaminoglycan inhibits thrombosis without affecting hemostasis via disrupting FIXa-FVIIIa complex generation

Lisha Lin [a, 1], Debing Pu [a, 1], Nanyu Xiong [a, b, 1], Dong Meng [a, d], Zhongtang Li [e], Xi Gong [a, b], Ya Li [a], Jiangyan Chen [a], Jiang Li [a], Lian Yang [a], Chuang Xiao [g], Chan Zhang [f], Xudong Dong [f], Mingyi Wu [a, b, c]*

[a] State Key Laboratory of Phytochemistry and Natural Medicines, Kunming Institute of Botany, Chinese Academy of Sciences, Kunming 650201, China.
[b] School of Traditional Chinese Pharmacy, Shenyang Pharmaceutical University, Shenyang 117004, China.
[c] University of Chinese Academy of Sciences, Beijing 100049, China.
[d] School of Pharmacy, Dali University, Dali 671003, China.
[e] State Key Laboratory of Natural and Biomimetic Drugs, School of Pharmaceutical Sciences, Peking University, Beijing, 100191, China.
[f] The First People's Hospital of Yunnan Province, The Affiliated Hospital of Kunming University of Science and Technology, Kunming 650032, China
[g] School of Pharmaceutical Science and Yunnan Key Laboratory of Pharmacology for Natural Products, Kunming Medical University, Kunming 650500, China.

[1] These authors contributed equally to this work.

*Mingyi Wu.
Email: wumingyi@mail.kib.ac.cn

1. Materials and methods

1.1 Materials

The snail *C. Cicatricosa* were purchased from Shaoguan city of Guangdong province, China. D-glucose (D-Glc), D-glucuronic acid (D-GlcA), N-Acetyl-D-galactosamine (D-GalNAc), D-galactose (D-Gal), D-galacturonic acid (D-GalA), ADP and lipopolysaccharide were from Sigma (USA). N-acetyl-D-glucosamine (D-GlcNAc) and D-Mannose (D-Man) were from Alfa Aesar (USA). L-iduronic acid (L-IdoA) was from Carbosynth (UK). L-Fucose (L-Fuc) was from TCI (Japan). 1-phenyl-3-methyl-5-pyrazolone (PMP) was from Aladdin (China). Human coagulation control plasma, APTT, PT, TT and $CaCl_2$ reagents were all from MDC Hemostasis (Germany). Anti-IIa kit (2 Stages Heparin Assay), Anti-Xa kit (2 Stages Heparin Assay), FVIII: C kit, antithrombin (AT), coagulation factor XIa and its substrate CS-21(66), coagulation factor XIIa and its substrate CS-31(02), FXa substrate CS-11(65) and thrombin were from Hyphen BioMed (France). Recombinant human FVIII was from Advate (Australia). Coagulation factor IXa, Xa and X were from Prolytix (USA), FIXa substrate was from Pentapharm (Switzerland). Amine-PEG3-Biotin was from Thermo Fisher Scientific (USA). Mouse IL-6 and TNF-α ELISA kits were from Invitrogen (USA). Phospholipid (PS: PC=3: 7) and hirudin were from Botaman (China). OSCS was from the National Institute for the Control of Pharmaceutical and Biological Products (China). Enox was from Sanofi (Fance). HS and Hep were kindly provided by DC Pharma (China). Other chemicals were of reagent grade and obtained commercially.

1.2. Animals and biological samples

The snail *C. cicatricose* was purchased from Shixing county (Shaoguan, China). Male SD rats (8–10 week) and male C57BL/6J mice (8–10 week) were from Institute of Medical Biology Chinese Academy of Medical Sciences and Beijing Sipeifu Biotechnology Co., Ltd. Animal experiments were approved by the Research Ethics Committee of the Kunming Institute of Botany, Chinese Academy of Sciences (kib202405012), and abided by National Research Council's Guide for the Care and Use of Laboratory Animals.

Rat blood was collected from inferior vena cava (IVC) at the end of DVT and A-V shunt experiments, and mouse blood was collected from orbital venous plexus at the end of endoxemia-enhanced DVT experiment. Nine volumes of blood sample were anticoagulated with one volume of 3.8% sodium citrate. Plasma samples were obtained after centrifugation (1000 g, 15 min), stored at -80℃ or assayed immediately.

1.3 Extraction and purification of CCG

Polysaccharides were isolated as previously reported with some modifications (1). In brief, the dried snail gastropods were ground into powder and defatted with anhydrous ethanol. Then, 10 volumes of deionized water and 2% alkaline protease were added, and incubated for 36 h (60°C and pH 9). After emzymolysis, the supernatant was obtained and its pH was adjusted to neutral, added 95% ethanol to a final concentration of 80%, then crude polysaccharide precipitate was obtained by centrifugation (4000 rpm, 15 min). The protein in crude polysaccharide was removed by trichloroacetic acid, then mucopolysaccharides were obtained by adding 10% benzethonium chloride. After decolorization with 3% H_2O_2, the mucopolysaccharides were further purified by FPA98 chromatography, eluting by NaCl solution, and the fraction of 0.8 M NaCl elution was concentrated, desalted and dried to obtain CCG.

1.4 Molecular weight and UV analysis

Molecular weight of CCG was determined by high-performance gel permeation chromatography (HPGPC) using an Agilent technologies 1260 series apparatus (USA) equipped with RID, DAD detectors and a Shodex OH-pak SB-804 HQ column (8 × 300 mm), analyzed by GPC 3.4.1 software and calculated from the standard curve. The standard curve was established by D-series dextrans standard (180, 2700, 5250, 9750, 13050, 36800, 646500, 135350, 300600 Da and 2000 kDa), fitted with a third-order polynomial [$LogMw = 10.96-0.501\ Rt + 0.006049\ Rt^2-0.00004485Rt^3$ ($R^2 = 0.9999$)], and calibrated by a heparin standard (16200 Da). The ultraviolet absorption of CCG sample was detected in 195–400 nm wavelength for the presence of protein or nuclei acid impurities, using UV-2450 spectrophotometer (Shimadzu, Japan).

1.5 Monosaccharide composition

The monosaccharide composition was analyzed by reverse-phase HPLC after PMP derivatization (2,3). CCG (1 mg/ml) was incubated with 4.0 M trifluoroacetic acid (TFA) at 110℃ for 4 h. After hydrolysis, the remnant TFA was removed with methanol under reduced pressure. Then the monosaccharide sample was reacted with 0.4 ml PMP (0.5 M in methanol) and 0.2 ml NaOH (0.6 M) at 70℃ for 1 h, the pH was adjusted to 7.0 and the remnant PMP was removed by chloroform. PMP derivatives were analyzed by Agilent technologies 1260 series high performance liquid chromatography (HPLC) equipped with DAD detectors and Hadesil C18-BIO column (4,5).

1.6 Methylation analysis

Methylation analysis of CCG was conducted as previously reported (1). Briefly, CCG (100 mg) was subjected to a hydrogen-type cation exchange column and dried to give a full hydrogen-type CCG. Then, the sample was added with 5 ml 0.1 M morpholinoethanesulfonic acid (MES), adjusted pH to 4.7 by triethylamine, mixed with 20 mg 1-ethyl-(3-Dimethylaminopropyl)-3-carbodiimide (EDC) for 1 h, then added 4.0 ml 4.0 M $NaBH_4$, after 30 min added another equal $NaBH_4$ and reacted for 1 h at 50℃, stopped by acetic acid. The sample was dialyzed with a 500 Da cut-off membrane, and lyophilized. The reduction procedures were repeated twice.

The reduced sample (3.0 mg) was added in 1.0 ml anhydrous DMSO at 60℃ for 1 h, then cooled to room temperature. The sample in ice-bath was added with l.0 ml NaOH (50 mg/ml in DMSO) and 1.0 ml CH_3I, and reacted at 25℃ for 1 h (in dark, sealing and ultrasound conditions), stopped by adding 2 ml deionized water, and the sample was dialyzed with a 500 Da cut-off membrane and dried under decompression to give methylated products.

Furthermore, the methylated products were hydrolyzed by 2.0 M TFA at 120°C for 2 h, then TFA was removed. The hydrolyzed product was reduced by 0.1 ml 0.1 M NaOH and 15 mg $NaBD_4$ (50°C, 2 h), stopped by 100 μl acetic acid. The sample was further reacted with 0.5 ml acetic anhydride and 0.5 ml pyridine (40°C, 2 h, under ultrasound), stopped by 1 ml deionized water, and the sample was extracted by 2 ml CH_3Cl for 3 times to obtain the partially methylated alditol acetates (PMAAs), which were analyzed by GC–MS on an Agilent HP6890GC/5973 MS system.

1.7 Analysis of the branched structure in CCG

The branches of CCG were partially removed by mild acid hydrolysis. 35.0 mg CCG was reacted with 7.0 ml 0.1 M H_2SO_4 at 80°C, 1.0 ml solution was taken out at appointed time and neutralized with $BaCO_3$ (6-8). The samples were centrifugated (3500 g, 10 min), the supernatant (0.6 ml) was filtrated by 0.2 μm membrane, and analyzed by HPLC with Shodex SB-804 HQ (8.0 × 300 mm) or Superdex 30 Increase 10/300 GL column. For the remaining supernatants (0.3 ml), polysaccharides were removed by 90% ethanol precipitation, then monosaccharides were subjected to PMP derivatization and HPLC analysis (Hadesil C18-BIO column). Other 200.0 mg of CCG was reacted under the above conditions for 3 h, and then treated with $BaCO_3$. The supernatant was precipitated with 90% ethanol. The obtained precipitate (CCG-MC) and supernatant (CCG-SC) were analyzed by NMR.

1.8 Depolymerization of CCG

To further elucidate the structure of CCG in detail, its depolymerized products were prepared (1). 350 mg CCG and 0.9 mg cupric sulfate were dissolved in 12.8 ml deionized water, which were mixed with 0.4 ml 30% H_2O_2 solution, and incubated at 35°C for 1 h. After reaction, the depolymerized product was precipitated with ethanol (final concentration of 80%), and the precipitate was dissolved in water, repeat this procedure for 3 times. Then the obtained depolymerized product (dCCG) was dialyzed with a 500 Da cut-off membrane for 24 h, and lyophilized.

1.9 NMR analysis

Structure of polysaccharides were analyzed by NMR technique using 800 MHz Bruker Avance spectrometer equipped with a ^{1}H-^{13}C dual probe in FT mode (1). The ^{1}H-^{1}H correlated spectroscopy (COSY), ^{1}H-^{13}C heteronuclear single-quantum coherence (HSQC), total correlation spectroscopy (TOCSY), heteronuclear multiple bond coherence (HMBC), and rotating frame overhauser effect spectroscopy (ROESY) spectra were recorded. All chemical shifts were normalized with internal 3-trimethylsilyl-(2,2,3,3-d_4)-propionic acid sodium (TSP-d_4, δ_H and δ_C = 0.00).

1.10 Anticoagulant activity

Coagulation time, including activated partial thromboplastin time (APTT), prothrombin time (PT), and thrombin time (TT), was determined using a coagulometer (TECO MC-4000, Germany). Compounds were dissolved and diluted with Tris-HCl (0.02 M, pH 7.4) buffer. Compound plasma concentration-clotting time was fitted linearly with GraphPad Prism, and $EC_{2.0\times}$ (concentration required for doubling clotting time) were calculated.

1.11 Thromboelastogram (TEG)

After anesthesia, blood was collected from C57BL/6J mice by cardiac puncture, anticoagulated with 3.8% sodium citrate (1: 9), then blood sample was used for thromboelastogram study. 360 μl blood was incubated with 3.6 μl compound solution or vehicle for 2 min, then 340 μl treated blood was added with 20 μl $CaCl_2$ (0.2 M) solution, and the thromboelastogram was recorded using a thromboelastometer (BVCA-1, Baorui, China).

1.12 Coagulation factor activity

The activity of thrombin (FIIa), FXa, FIXa, FXIa and FXIIa was determined by chromogenic substrate method using the coagulation factor and its substrate, the optical density (OD) was measured at 405 nm using a microplate reader (Bio-Tek ELx 808, USA). For anti-iFXase assay, Biophen FVIII:C kit (containing R1/FX, R2/FIX, and R3/FXa substrate) and FVIII were used. For anti-FIIa and anti-FXa assay, Biophen anti-IIa kit (containing R1/AT, R2/FIIa, and R3/FIIa substrate) and anti-Xa kit (containing R1/AT, R2/FXa, and R3/FXa substrate) were used, respectively. The change rate of absorbance at 405 nm (OD_{405nm}/min) in the presence of test compound was normalized to that of the control. Compound concentration - relative activity was plotted and fitted (log(inhibitor) *vs.* response -- Variable slope (four parameters)) with GraphPad Prism, and IC_{50} values were calculated.

1.13 Biolayer interferometry (BLI)

Biotin labeling: glycosaminoglycan was reacted with Amine-PEG3-Biotin, with the molar ratio of 2: 1, at 70℃ for 24 h, then reacted with $NaBH_3CN$, with the molar ratio of 1000:1, at 70℃ for 24 h. The biotinylated product (Bio-CCG or Bio-Hep) was purified by a Zeba™ spin desalting column (7 kD cut-off, Merck, Germany).

CCG-protein interaction: Bio-CCG was immobilized onto the surface of streptavidin (SA) biosensors. FIXa at 40 nM or FVIIIa at 40 nM was added in the black 96-micoplate to interact with the immobilized CCG. The association and dissociation processes were set as 300 s, and the binding signals were detected using an Octet Red 96 instrument (Fortebio, USA). Biotinylated CCG or heparin was immobilized onto SA biosensors. FIXa at a range of concentrations of 6.25, 12.5, 25, 50, 100 and 200 nM were added in the black 96-micoplate to interact with the immobilized CCG or Hep. After the ligand on sensors was regenerated with 10 mM Glycine-HCl, AT at a series of concentrations of 62.5, 125, 250, 500, 1000 and 2000 nM were added in the black 96-micoplate to interact with the immobilized CCG or Hep. The association and dissociation processes were set as 900 s. The BLI binding signals were analyzed using the Octet 7.0 software and the binding curves were globally fitted by a 1:1 model.

1.14 Determination of FVIIIa decay rate and apparent affinity of FIXa-FVIIIa

The effects of CCG on FVIIIa activity and the affinity of FIXa-FVIIIa were conducted, according to previous reported methods. FVIIIa activity-time data, with or without CCG, were fitted by exponential equation, and the observed first-order rate constant (k_{obs}) was calculated from $V_t=V_0e^{-K_{obs}t}$, where V_t and V_0 was FXa generation rate at time t and 0, respectively, and e was the natural constant. The apparent affinity ($K_{D(app)}$) of FIVa-FVIIIa, with or without CCG, was calculated from $B=B_{max}C/(K_{D(app)}+ C)$, where, B was the FXa generation rate under different

concentrations of FIXa, B_{max} was the FXa generation rate under saturated concentration of FIXa, and C was the concentration of FIXa.

1.15 Plasma contact activation

The effect of compound on plasma contact activation was detected as previously reported. The generated FXIIa was assessed by its cleavage of chromogenic substrate D-Pro-Phe-Arg-pNA, detected at OD_{405nm} every 15 s for 2 min at 37°C. Compound concentration - activity was plotted by Origin 2021.

1.16 Platelet aggregation

Blood was collected from rabbit central ear artery, and anticoagulated with 3.8% sodium citrate, after centrifugation (200 g, 8 min), the platelet rich plasma (PRP) was collected for platelet aggregation assay.

1.17 In vivo antithrombotic studies

Rat DVT experiment: Thrombosis was induced by a combination of stasis and thromboplastin (9). After anesthesia, rat midline laparotomy was performed, IVC was exposed, and a loose suture was put under the IVC next to left renal vein. Compound was injected intravenously (CCG was at 5, 10 and 20 mg/kg, HS was at 1.25 mg/kg, Enox was at 0.8 mg/kg, and Hep was at 0.4 mg/kg), after 10 min, 2% thromboplastin suspension (1 ml/kg) was injected and the suture was lightened. After 20 min thrombus was collected, its length and weight were measured. Rat blood was collected and anticoagulated, and plasma samples were prepared for APTT, PT and TT assays.

Rat A-V shunt model: After anesthesia rat left external jugular vein and right carotid artery were exposed, a catheter containing a 4-0 suture were prepared and filled with saline, the ends of catheter were inserted into the exposed vein and artery, respectively, to form a A-V shunt (10). The A-V shunt circulation was opened 10 min after compound injection (CCG was at 10 mg/kg, HS and Enox was at 5 mg/kg). After 15 min, thrombus was taken from the A-V shunt and weighed, dry thrombus was weighed after drying at 60℃ for 1 h. Rat blood was collected and anticoagulated, and plasma samples were prepared for APTT, PT and TT assays.

Mouse endotoxemia-enhanced DVT model: After compound injection (CCG was at 5 and 10 mg/kg, Hep was at 1 mg/kg) for 10 min, mice were anesthetized, and midline laparotomy was performed to expose IVC, and a loose suture was put under the IVC next to left renal vein. Lipopolysaccharide (10 mg/kg) was intravenously injected, and IVC was ligated (11). After 6 h, mouse blood was collected and anticoagulated, mice were euthanized, and the thrombus (if presented) was measured and weighed (dry thrombus was weighed after drying at 60℃ for 1 h). Plasma samples were prepared for IL-6 and TNF-α ELISA assays.

Mice tail bleeding assay: After compound injection (CCG was at 70 mg/kg, HS was at 8.76 mg/kg, Enox was at 5.6 mg/kg, and Hep was at 2.8 mg/kg) for 10 min, mice were anesthetized, and 5 mm segment of the distal tail was cut (9). The tail was immediately immersed in 8 mL pure water (37℃) for 1 h, and the solution was refilled with pure water to 10 ml. The hemoglobin in the hemolyzed blood solution was measured at 540 nm using a using a microplate reader. Volume of blood loss was calculated from the OD_{540nm} -blood volume standard curve.

1.18 Modelling study

Crystal structure of DTRI-177-FIXa complex (PDB ID: 8EPK) was selected for the study. The structure was prepared by the Protein Preparation Wizard module in Schrodinger 10.2 software (NY, USA). After removing DTRI-177, FIXa was assigned bond orders, added hydrogens, protonated, removed all crystallographic water molecules,

and restrained minimization. The structure of CCG pentasaccharide unit was prepared by the LigPrep module. Then, energy-minimized conformations were docked into the binding site defined Arg165 and Arg233. Three predicted binding pose was generated (Maestro 11.5).

1.19 Statistical analyses

The data were analyzed by GraphPad Prism, for data of three or more groups one-way ANOVA followed by Fisher's LSD Test or Kruskal-Wallis test (when data is not normally distributed) was used, for data of two groups unpaired two-tailed t-Test was used. Compared with the control or model group, P values less than 0.05 were considered statistically significant.

2. Structural characterization of CCG

2.1 Analysis of the branched structure in CCG

Methylation analysis indicated that galactose in CCG is present only in the form of terminal linkages, suggesting it may be existed as a side chain. Since side chains in polysaccharides are more susceptible to acid hydrolysis than main chains (12-13), mild acid hydrolysis was conducted to obtain the branches (Fig. S2A). As analyzed by a Superdex Peptide10/300 GL column, the amount of a new low-molecular-weight product was gradually increased with the extension of hydrolysis time, and reaching the highest level at 180 min (Fig. S2B, D -E). The new product was indicated to be galactose, according to PMP-derivatization and NMR analysis (Fig. S2D and Fig. S3). Therefore, CCG possesses a unique side chain of single galactose. Meanwhile, the retention time of remaining CCG (high-molecular-weight fraction) gradually increased on OHpak SB-804 HQ column, indicating that its molecular weight is slowly decreasing. (Fig. S2C). After hydrolysis for 180 min, the obtained CCG main chain (CCG-MC) had a retention time of 16.0 min (Fig. S4A), corresponding to 19.2 kDa (PD = 1.8) by GPC analysis (Fig. S4B, Table S1). In ^{1}H NMR (Fig. S5), the signals of CCG-MC were distinctly different from those of CCG, but their signals were both more complex than those of heparin and heparan sulfate, suggesting that their more intricate structures.

2.2 Glycosidic linkage analysis

Methylation and GC-MS analysis (Fig. S6A) showed that CCG derivatives contained four PMAAs and four non-methylated acetate derivatives (Fig. S6B–C). According to the database GC-EIMS (Complex Carbohydrate Research Center, University of Georgia), four PMAAs derived from CCG were determined to be 1,5-di-*O*-acetyl-1-deuterio-2,3,4,6-tetra-*O*-methyl-D-galactitol (**1**), 1,4,5-tri-*O*-acetyl-1-deuterio-2,3,6-tri-*O*-methyl-D-glucitol (**2**), 1,4,5-tri-*O*-acetyl-1-deuterio-2-*N*-acetyl-2-*N*-methyl-3,6-di-*O*-methyl-D-glucitol (**3**), and 1,3,4,5-tetra-*O*-acetyl-1-deuterio-2-*N*-acetyl-2-*N*-methyl-6-*O*-methyl-D-glucitol (**4**) (Fig. S6C and Fig.S7). In which, PMAA (**1**) was assigned to terminal-linked Gal, suggesting a galactose monosaccharide side chain in CCG. PMAA (**2**) was attributed to 1,4-linked Glc (or Ido). Since carboxyl group of the acid monosaccharide was reduced to a primary hydroxyl prior to methylation, the 1,4-linked Glc should be assigned to 1,4-linked-GlcA and/or 1,4-linked-IdoA in CCG. PMAA (**3**) was appointed to 1,4-linked GlcNAc, and PMAA (**4**) was attributed to 1,3,4-linked GlcNAc, indicating that the terminal-linked Gal may link to the 3rd position of GlcNAc. Hence, the glycosidic linkages within CCG mainly contained terminal-linked-Gal, 1,4-linked-GlcNAc, 1,4-linked-GlcA, 1,4-linked-IdoA and 1,3,4-linked-GlcNAc (Fig. S6D).

2.3. NMR analysis

Detailed structure of CCG was characterized by NMR analysis. The NMR signals of CCG were seriously

overlapped, therefore, a depolymerized CCG (dCCG) was prepared by free radical depolymerization. The HPGPC of dCCG showed a singlet at 17.2 min, corresponding to the molecular weight of 7.0 kDa (Fig. S8, Table S1). The chemical shift signals of dCCG and CCG in NMR spectra were similar (Fig. S9), indicating that the depolymerization process hardly change the main structural features of CCG. While the signals of dCCG were clearer, enabling structural characterization more accurate.

In the ^{1}H NMR spectrum (Fig. S9A), the typical signal at δ_H 2.07 ppm can be assigned to the proton of methyl group (-COC$\underline{H}_3$) in GalNAc. The characteristic signals at δ_H 4.53 and δ_H 3.42 ppm were primarily appointed to anomeric proton (H-1) and H-2 of β-GlcA, respectively. The signals in δ_H 3.00–3.20 ppm were assigned to the protons (H-2) of GlcNS in different chemical environments. The signals in δ_H 3.60–4.30 ppm were primarily ascribed to the protons of sugar residues, which were highly overlapped. Compared with heparin and heparan sulfate (Fig. S5), CCG showed multiple anomeric proton signals in low-field region (above 5.0 ppm), implying that its complex structure.

For ^{13}C NMR spectrum (Fig. S9B), the signal around δ_C 25 ppm was attributed to methyl group (-CO$\underline{C}H_3$) in GlcNAc, and signals in δ_C 55-58 ppm were assigned to C-2 of GlcNAc, which shifted upfield after aminoation. In DEPT spectrum, the signals between δ_C 60-65 ppm appeared negative peaks, assigned to methylene of oxidized C-6 in sugar residues. Several distinct anomeric carbon signals between δ_C 97-105 ppm could be observed. The signals between δ_C 175-180 ppm were assigned to carboxyl at C-6 of uronic acid and acetyl group (-$\underline{C}OCH_3$) in GlcNAc.

The 1D NMR signals of dCCG were also heavily overlapped, thus its structural elucidation mainly relied on the comprehensive analysis of 2D NMR correlation spectra (Fig. S10). In the HSQC spectrum, there were mainly nine cross-peaks in the anomeric signal region, which are δ 4.53/105.3, 5.04/97.2, 5.06/103.3, 5.09/97.1, 5.12/101.8, 5.16/99.3, 5.28/101.4, 5.40/101.8, 5.46/99.9 ppm, indicating that dCCG contained nine types of monosaccharide residues in different chemical environments. Among them, the anomeric signal of residues A was relatively isolated at δ 4.53/105.3 ppm, of which ^{1}H chemical shift was at higher field, significantly less than δ_H 5.0 ppm. Typically, in NMR spectroscopy, the chemical shift of anomeric proton in a β-configuration monosaccharides appeared upfield relative to that of α-configuration, usually less than 5.0 ppm (14). Thus, it's speculated that A is a β-configuration, while other residues are α-configuration.

For residue A, in the ^{1}H-^{1}H COSY correlation spectrum, 4.53 (H-1)/3.42 (H-2) and 3.42 (H-2)/3.76 (H-3) ppm were cross-correlated, indicating that the chemical shifts of H-2 and H-3 are 3.42 and 3.76 ppm, respectively. Furthermore, through 3.91 (H-5)/105.3 (C-1) ppm HMBC heteronuclear long-range correlation, 4.53 (H-1)/3.81 (H-4) TOCSY long-range correlation, and 3.42 (H-2)/ 3.81(H-4) ROESY spatial correlation, it was determined that the chemical shifts of H-4 and H-5 are 3.81 and 3.91 ppm, respectively. The chemical shifts of C-2 to C-5 on the sugar ring were determined as: 75.9 (C-2), 78.2 (C-3), 80.9 (C-4), and 80.0 (C-5) ppm through HSQC correlation signals. In HMBC correlation spectrum, through the correlation signal of 3.81 (H-4) with 177.4 (C-6), the chemical shift of C-6 was assigned as 177.4 ppm, thus determining residue A as an β-configured uronic acid. In addition, H-1/H-2, H-1/H-3, H-2/H-3, and H-2/H-4 TOCSY long-range correlations, H-2/C-1, H-2/C-3, H-3/C-1, H-3/C-2, H-3/C-4, H-4/C-3, H-4/C-5, and H-5/C-1 HMBC correlations, H-2/C-1, H-2/C-3, H-2/C-4, H-2/C-5, H-3/C-1, H-3/C-5, and H-4/C-5 HSQC-TOCSY correlations collectively provided further confirmation of the chemical shift assignments for A (Fig. S10, Table S2). Compared with the NMR data of fondaparinux sodium (15-16), the chemical shifts of A was

consistent with the signals of 1,4-linked β-D-GlcA, thus residue A was determined as →4-D-GlcA-1β→.

For residue B, through continuous COSY correlations, the chemical shifts of H-1 to H-5 were determined to be 5.04, 4.26, 3.94, 3.70, and 3.88 ppm, respectively. By the ROESY correlation signal between H-2 and H-6, the chemical shift of H-6 was determined as 3.88 ppm, overlapping with the peak of H-5. Combined with the HSQC correlation signal, the chemical shifts of C-1 to C-6 in B were determined to be 97.2, 56.2, 75.8, 77.0, 74.4, and 62.3 ppm, respectively. The chemical shift of C-2 at 56.2 ppm was upfield, which could be attributed to the influence of N-acetylamino substitution at C-2. In the HMBC spectrum, H-2 and H-8 cross-correlated with C-7, indicating that the acetylamino group is connected to C-2. Thus, B is characterized as α-D-GlcNAc (Fig. S10, Table S2). However, compared with the NMR data of 1,4-linked α-D-GlcNAc (1), the chemical shifts of H-3 and C-3 in B both shifted to a lower magnetic field, suggesting that there may be another sugar moiety (E unit) linked to C-3 of B. In addition, there were another set of signals (residue B') similar to that of B (Fig. S10, Table S2), with only minor differences at the anomeric position (B, δ_H 5.04, δ_C 97.2 ppm; B', δ_H 5.16, δ_C 99.3 ppm). It's preliminarily speculated that two distinct sugar residues (C and C' units) linked to the first site (C-1) of B, leading to different chemical environment at the anomeric position and its adjacent sites of B and B'.

Similarly, through the comprehensive analysis of COSY, TOCSY, ROESY, HSQC, HMBC, and HSQC-TOCSY 2D correlation signals (Fig. S10, Table S3), it indicated that sugar residue C is another α-configured aldonic acid. Besides, C and C' showed signals with similar chemical shifts. The differences between C and C' were mainly in the 2nd (C-2, δ_H 4.55, δ_C 74.1 ppm; C-2', δ_H 4.53, δ_C 79.9 ppm), 3rd (C-3, δ_H 4.78, δ_C 71.8 ppm; C-3', δ_H 4.23, δ_C 69.2 ppm), and 4th (C-4, δ_H 4.22, δ_C 71.6 ppm; C-4', δ_H 3.75, δ_C 78.1 ppm) positions of the sugar ring. Compared with the NMR data of the fondaparinux sodium derivative (15), the signals of C was consistent with that of 1,4-linked α-L-IdoA2S3S, and C' with 1,4-linked α-L-IdoA2S. Thus, C was determined as →4-L-IdoA2S3S-1α→, and C' as →4-L-IdoA2S-1α→.

Similarly, residue D was determined to be α-D-amino sugar (Fig. S10, Table S2). Sets of similar signals were also observed, which were mainly assigned to three obvious sets, D, D', and D'' (Table S2), with significant differences in the chemical shifts of 2nd and 3rd positions. According to literature (15–16), the high-field signal at δ_H 3.1 ppm in hydrogen spectrum, was assigned to 2nd proton of α-D-GlcN2S, determining that D and D' were sulfated glucosamine at 2nd position. The signals at 2nd position of D'' (δ_H 4.01) were similar to that of α-D-GlcNAc, determining that D'' is acetylated glucosamine. The proton signal (δ_H 4.59 ppm) at 3rd position of D' was significantly more downfield than that of D and D'' (δ_H 3.90 and 3.88 ppm, respectively), indicating that 3rd position of D' may be substituted by a sulfate group. Taken together, D, D', and D'' were determined as →4-D-GlcN2S-1α→, →4-D-GlcN2S3S-1α→, and →4-D-GlcNAc-1α→, respectively.

By analysis, residue E was determined as terminal-substituted α-galactose (Gal-1α→), and its chemical shift assignments were shown in Table S1.

The basic residues in dCCG determined by NMR analysis were consistent with the results of monosaccharide composition, methylation and mild acid hydrolysis, and their type and position of substituents were also clarified (Table S2). The residues linkage order was mainly assigned by HMBC and ROESY signals (Fig. 2C–D, Fig. S10E–F, Table S4). The HMBC correlation between H_{B4} and C_{A1}, and the ROESY correlation between H_{B4} and H_{A1},

indicated that 1st position of A was connected to 4th position of B by a glycosidic bond. The HMBC correlations of H_{B3}/C_{E1} and H_{E1}/C_{B3}, and the ROESY correlation of H_{B3} and H_{E1}, suggested that 1st position of E was linked to the 3rd position of B. The HMBC correlation between H_{C4} and C_{B1}, and the ROESY correlation between H_{C4} and H_{B1}, demonstrated that 1st position of B was connected to 4th position of C. The HMBC correlations of H_{C1}/C_{D4} and H_{D4}/C_{C1}, and the ROESY correlations of H_{C1}/H_{D4} and H_{D4}/H_{C1} showed that 1st position of C was linked to 4th position of D. The HMBC correlation between H_{A4} and C_{D1}, and the ROESY correlations of H_{D1}/H_{A4} and H_{A4}/H_{D1}, indicated that 1st position of D is connected to 4th position of A. In addition, the HMBC correlation between $H_{D''4}$ and $C_{C'1}$, and the ROESY correlations of H_{A2}/H_{E5}, H_{E5}/H_{A1}, $H_{C'1}/H_{D'4}$, $H_{D'4}/H_{C2}$, $H_{A4}/H_{D''1}$, and $H_{D4}/H_{C'1}$, further verified the glycosidic linkages type of residues. Therefore, the sequence of dCCG was determined as {→4)-A-(1→4)-[E(1→3)]-B-(1→4)-C(or C')-(1→4)-D(or D', or D'')-(1→}$_n$ (Fig. 2E), consistent with the results of methylation analysis.

The results have elucidated the structural details of a pentasaccharide (Fig. 1B), as the repeating unit for CCG. Notably, six possible pentasaccharide repeating units were proposed. Among them, due to the relatively high proportion of residues C and D, the first pentasaccharide unit is likely the predominating fragment constituting CCG. Thus, the polysaccharide parent could be determined as {→4)-D-GlcA-β(1→4)-[D-Gal-α(1→3)]-D-GlcNAc-α(1→4)-L-IdoA2S(or 2S3S)-α(1→4)-D-GlcN(2S, or 2S3S, or 2Ac)-α(1→}$_n$.

3. Supplementary references

1. Wu Y, *et al.* A non-anticoagulant heparin-like snail glycosaminoglycan promotes healing of diabetic wound. *Carbohydr Polym* 2020; **247**: 116682.
2. Liu D, Tang W, Yin J-Y, Nie S-P, Xie M-Y. Monosaccharide composition analysis of polysaccharides from natural sources: Hydrolysis condition and detection method development. *Food Hydrocoll* 2021; **116**: 106641.
3. Honda S, Akao E, Suzuki S, Okuda M, Kakehi K, Nakamura J. High-performance liquid chromatography of reducing carbohydrates as strongly ultraviolet-absorbing and electrochemically sensitive 1-phenyl-3-methyl-5-pyrazolone derivatives. *Anal Biochem* 1989; **180**: 351-357.
4. Zhang J, *et al.* Structures of fructan and galactan from *Polygonatum Cyrtonema* and their utilization by probiotic bacteria. *Carbohydr Polym* 2021; **267**: 118219.
5. Gu Q, *et al.* The structures of two glucomannans from *Bletilla Formosana* and their protective effect on inflammation via inhibiting NF-kappaB pathway. *Carbohydr Polym* 2022; **292**: 119694.
6. Wu M, *et al.* Structure and effect of sulfated fucose branches on anticoagulant activity of the fucosylated chondroitin sulfate from sea cucumber *Thelenata ananas*. *Carbohydr Polym* 2012; **87**: 862-868.
7. Pomin VH, Mourao PA. Specific sulfation and glycosylation-a structural combination for the anticoagulation of marine carbohydrates. *Front Cell Infect Microbiol* 2014; **4**:33.
8. Mourão PA, *et al.* Structure and anticoagulant activity of a fucosylated chondroitin sulfate from echinoderm. Sulfated fucose branches on the polysaccharide account for its high anticoagulant action. *J Biol Chem* 1996; **271**: 23973-23984.
9. Zhou L, *et al.* Effects of native fucosylated glycosaminoglycan, its depolymerized derivatives on intrinsic factor Xase, coagulation, thrombosis, and hemorrhagic risk. *Thromb Haemost* 2020; **120**: 607-619.

10. Shang J, *et al*. Zucker diabetic fatty rats exhibit hypercoagulability and accelerated thrombus formation in the arterio-venous shunt model of thrombosis. *Thromb Res* 2014; **134**: 433-439.
11. Obi AT, *et al*. Endotoxaemia-augmented murine venous thrombosis is dependent on TLR-4 and ICAM-1, and potentiated by neutropenia. *Thromb Haemost* 2017; **117**: 339-348.
12. Berman ER. Separation of connective tissue mucopolysaccharide-protein complexes from unrelated proteins. *Nature* 1966; **211**: 640-641.
13. Chen SG, Li GY, Ye XQ, Xue CH. Partial hydrolysis of the fucosylated chondroitin sulfate from sea cucumber *Isostichopus badionotus* and its mechanism. *Chinese J Struc Chem* 2012; **31**: 1455-1463.
14. Speciale I, *et al.* Liquid-state NMR spectroscopy for complex carbohydrate structural analysis: A hitchhiker's guide. *Carbohydr Polym* 2022; **277**: 118885.
15. Zhang GQ, *et al.* An efficient anticoagulant candidate: Characterization, synthesis and study of a fondaparinux analogue Rrt1.17. *Eur J Med Chem* 2017; **126**: 1039-1055.
16. Pomin VH. NMR Chemical shifts in structural biology of glycosaminoglycans. *Anal Chem* 2014; **86**: 65-94

4. Supplementary figures and tables

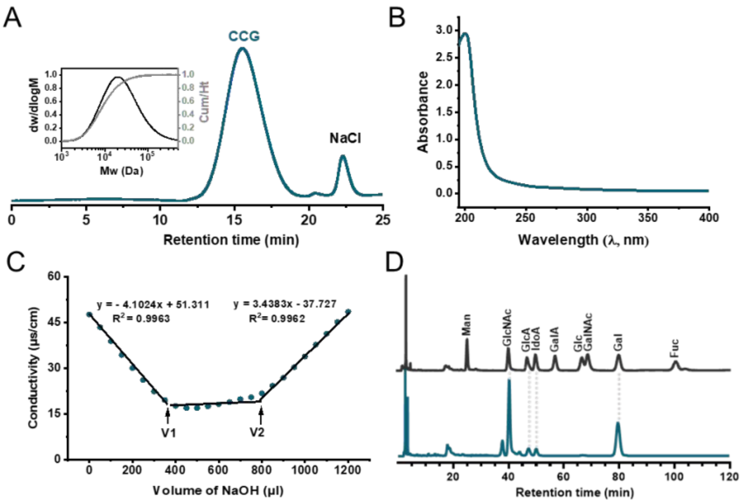

Fig. S1 Chemical characterization of CCG. (A) The high-performance gel permeation chromatography **(**HPGPC) profile of CCG. (B) Ultraviolet spectrum of CCG. (C) Conductimetric titration curve of CCG. (D) HPLC profile of monosaccharide PMP derivatives from CCG.

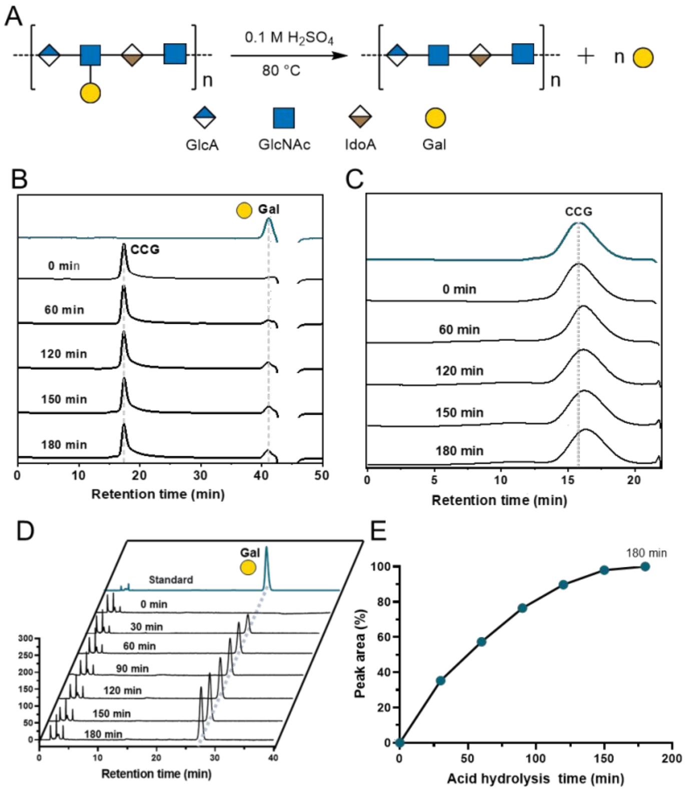

Fig. S2 Mild acid hydrolysis of snail glycosaminoglycan CCG. (A) The scheme of partial acid hydrolysis reaction for CCG. (B-C) HPGPC profiles of the acid hydrolysis products at different reaction time analyzed by Superdex Peptide10/300 GL column (B) or OHpak SB-804 HQ column (C). (D-E) HPLC profiles of PMP derivatives of standard Gal and the new product (low-molecular-weight product) from hydrolysis at different reaction time (D), and their peak area (E).

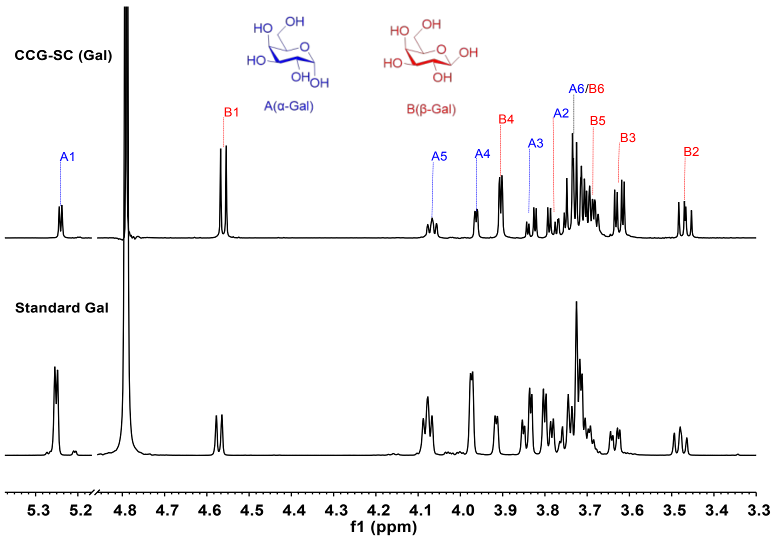

Fig. S3 ^{1}H NMR spectra of CCG side chain (CCG-SC) and standard galactose. CCG-SC was the low-molecular-weight new product resulting from hydrolysis. The letters represented α-galactose (A, blue) and β-galactose (B, red). The numbers indicated the positions of protons in the sugar ring.

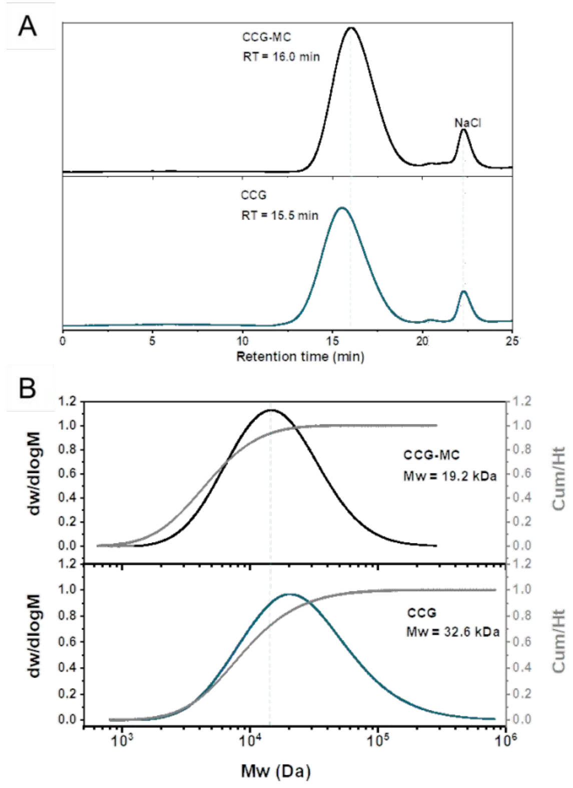

Fig. S4 HPGPC profiles of CCG main chain (CCG-MC) and CCG. (A) The HPGPC profiles were detected by Shodex TM OH-pak SB-804 HQ. (B) Molecular weight distribution curve of CCG-MC and CCG.

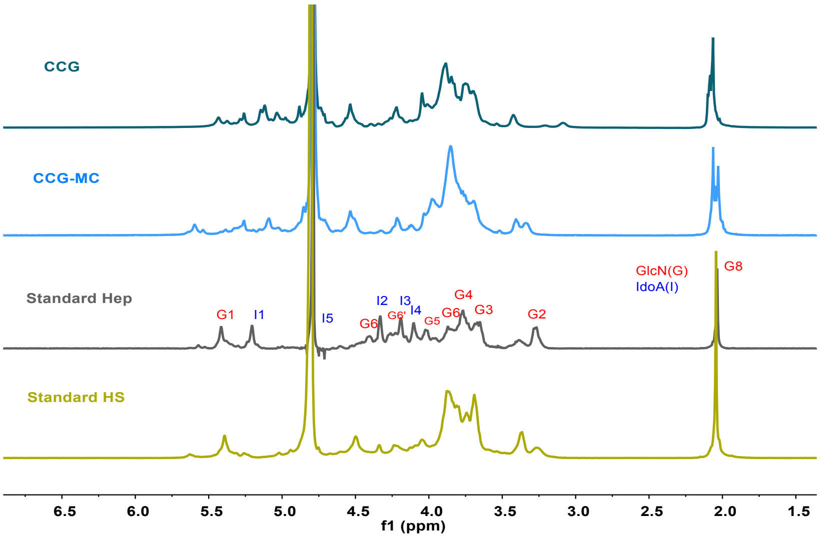

Fig. S5 ^{1}H NMR spectra of CCG, CCG-MC, heparin (Hep) and heparan sulfate (HS). Detected in D_2O.

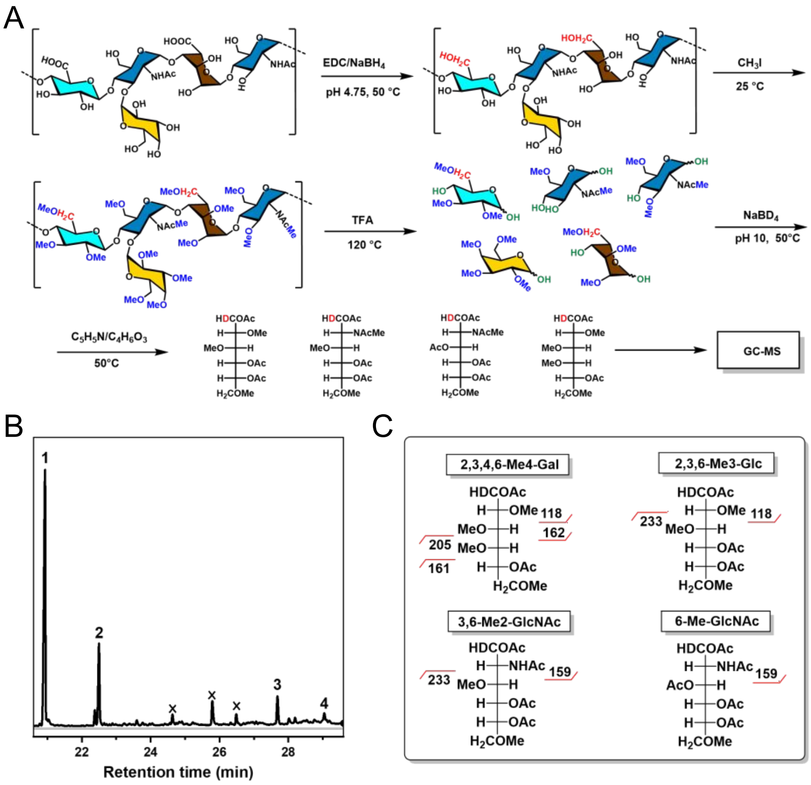

D

NO.	Methylated sugars	Type of linkages	Retention time (min)	Mass fragments (m/z)
1	2,3,4,6-Me_4-Gal	Terminal Gal	20.93	59, 71, 87, 88, 102, 118, 129, 145, 161, 162, 205
2	2,3,6-Me_3-Glc	1,4-linked Glc (or Ido)	18.87	71, 87, 99, 102, 113, 118, 129, 131, 233
3	3,6-Me_2-GlcNAc	1,4-linked GlcNAc	27.53	75, 87, 99, 117, 143, 159, 233
4	6-Me-GlcNAc	1,3,4-linked GlcNAc	28.82	58, 75, 117, 142, 159, 285

Fig. S6 Methylation analysis of CCG. (A) The scheme of methylation analysis for CCG. (B) Total ion chromatogram (TIC) profile of the partially methylated alditol acetates (PMAAs) derived from CCG. (C) Chemical

structures of alditol acetate derivatives. (D) GC–MS of alditol acetate derivatives. The noncarbohydrate signals are marked as ×.

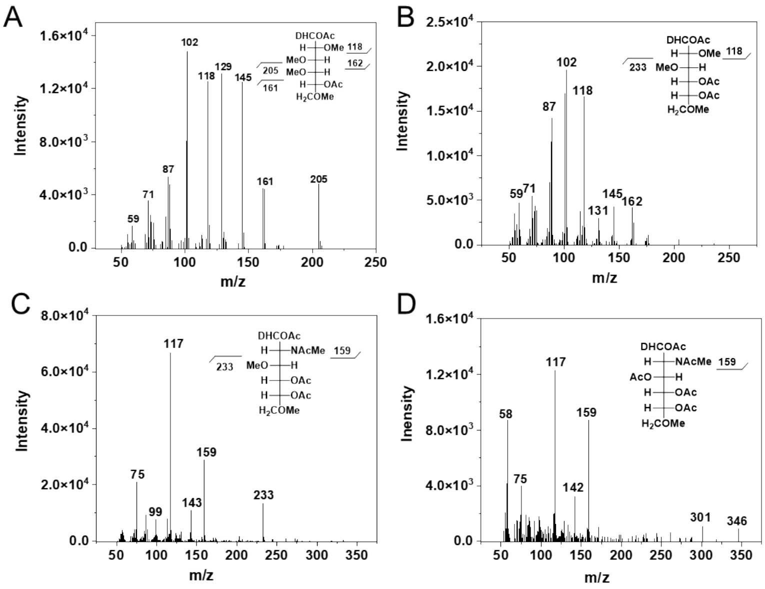

Fig. S7 MS fragments of PMAAs derived from CCG. (A) 1,5-Di-*O*-acetyl-1-deuterio-2,3,4,6-tetra-*O*-methyl-D-galactitol; (B) 1,4,5-Tri-*O*-acetyl-1-deuterio-2,3,6-tri-*O*-methyl-D-iditol; (C) 1,4,5-Tri-*O*-acetyl-2-(acetylmethylamino)-2-deoxy-1-deuterio-3,6-di-*O*-methyl-D-glucitol; (D) 1,3,4,5-Tetra-*O*-acetyl-2-(acetylmethylamino)-2-deoxy-1-deuterio-6-*O*-methyl-D-glucitol.

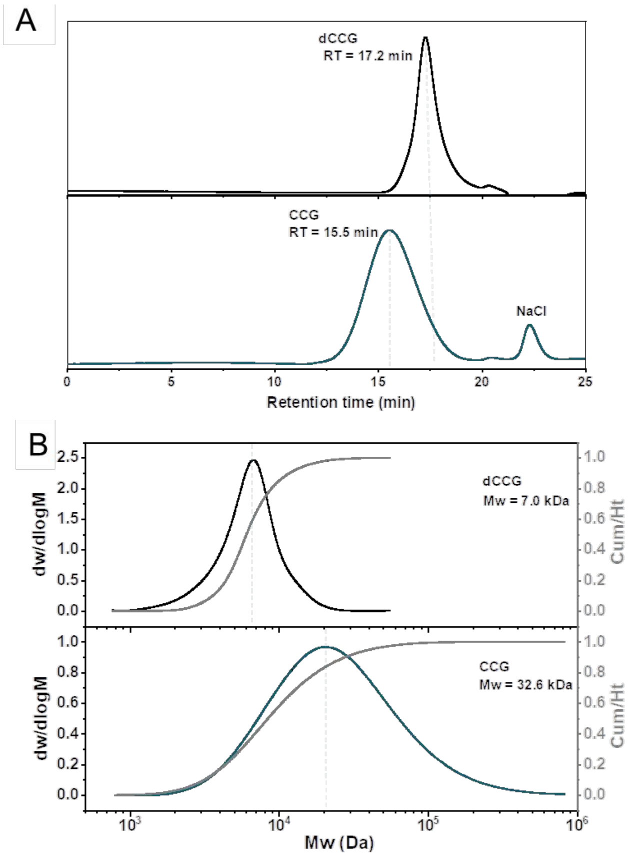

Fig. S8 The HPGPC profile and molecular weight of depolymerized CCG (dCCG) compared with CCG. (A) The HPGPC profiles were detected by Shodex TM OH-pak SB-804 HQ, dCCG was obtained from CCG by hydrogen peroxide depolymerization. (B) The molecular weight distribution curve of dCCG and CCG.

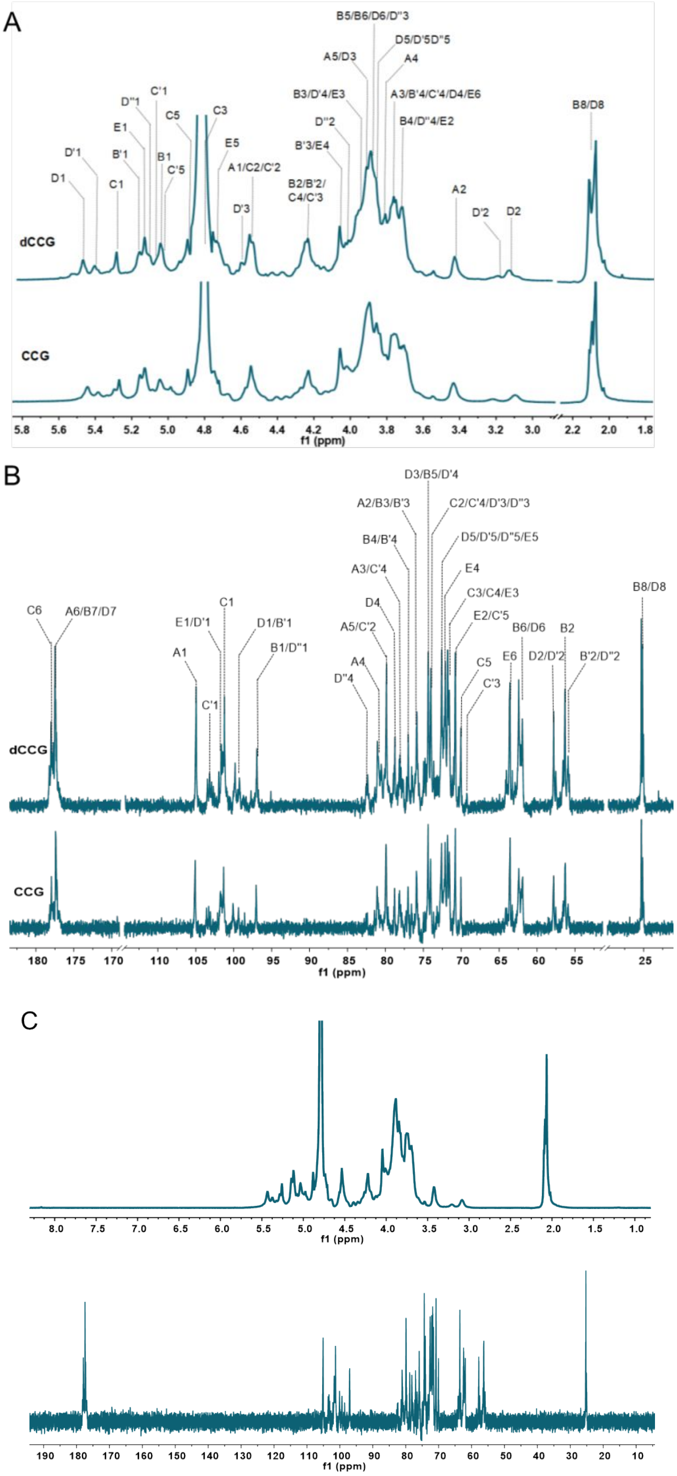

Fig. S9 ^{1}H and ^{13}C NMR spectra of dCCG and CCG. (A) ^{1}H NMR spectra of dCCG and CCG. (B) ^{13}C NMR spectra of dCCG and CCG. (C) The full spectra of CCG. The letters represented 1,4-linked glucuronic acid (GlcA, A), 1,3,4-linked *N*-acetylglucosamine (GlcNAc, B), 1,3,4-linked *N*-acetylglucosamine (GlcNAc, B'), 1,4-linked 2-

O-sulfated iduronic acid (IdoA2S, C), 1,4-linked 2,3-di-*O*-sulfated iduronic acid (IdoA2S3S, C'), 1,4-linked *N*-sulfated glucosamine (GlcNS, D), 1,4-linked 2,3-di-sulfated *N*-glucosamine (GlcN2S3S, D'), 1,4-linked *N*-acetylglucosamine (GlcNAc, D'') and terminal galactose (Gal, E), respectively, and the numbers indicated the positions of ^{1}H in the sugar residues. Detected in D_2O.

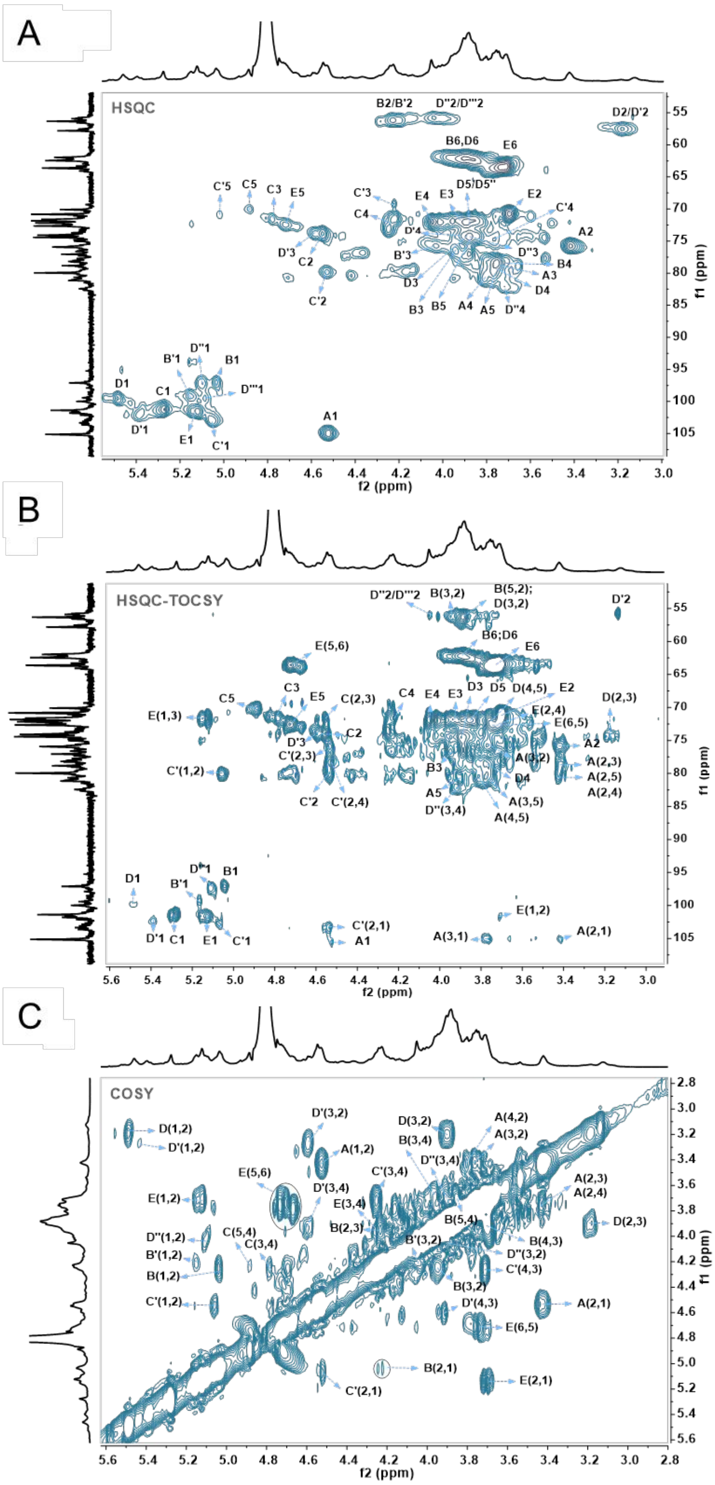

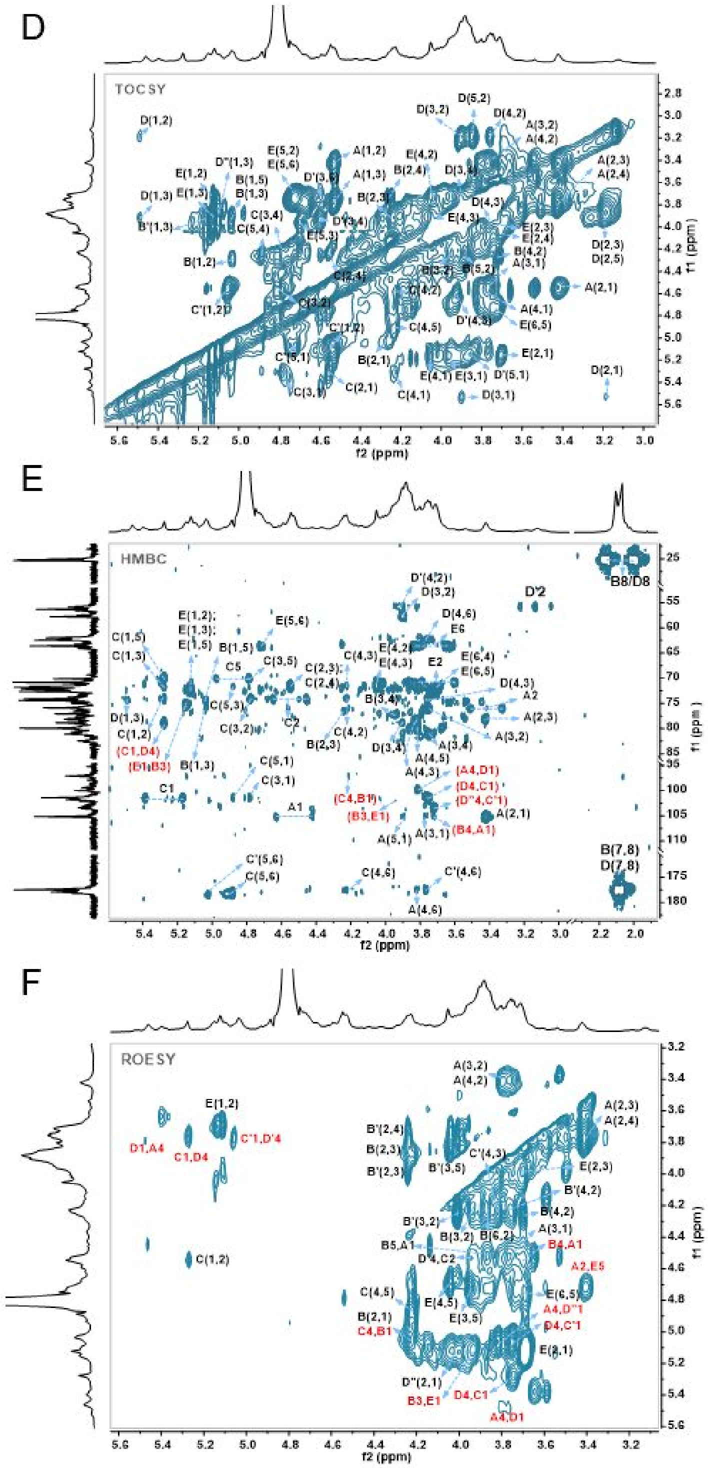

Fig. S10 2D NMR spectra of dCCG. (A) HSQC. (B) HSQC-TOCSY. (C) COSY. (D) TOCSY. (E) HMBC. (F) ROESY. The red marks in HMBC (E) and ROESY (F) indicated the ^{1}H-^{13}C correlation and spatial ^{1}H-^{1}H correlation of residue linkages, respectively. The letters were defined as follow: 1,4-linked glucuronic acid (A), 1,3,4-linked *N*-acetylglucosamine (B), 1,3,4-linked *N*-acetylglucosamine (B'), 1,4-linked 2-*O*-sulfated iduronic acid (C), 1,4-linked 2,3-di-*O*-sulfated iduronic acid (IdoA2S3S, C'), 1,4-linked *N*-sulfated glucosamine (D), 1,4-linked 2,3-di-sulfated *N*-

glucosamine (D'), 1,4-linked *N*-acetylglucosamine (D'') and terminal galactose (E). And the numbers indicated the positions of ^{1}H and ^{13}C in the sugar residues. Assays were conducted in D_2O.

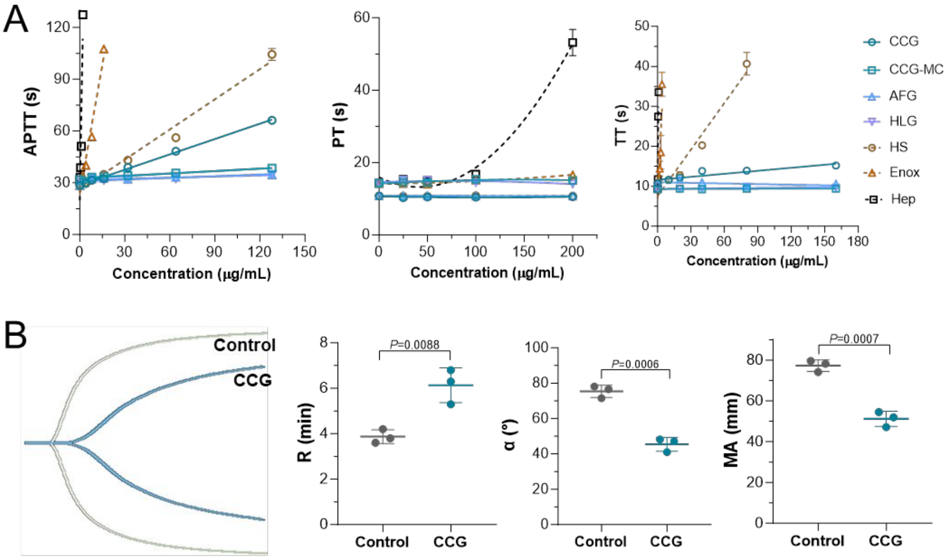

Fig. S11 The *in vitro* anticoagulant and antithrombotic activity of CCG. (A) The effect of snail GAGs on coagulation time of human plasma, including APTT, PT and TT. (B) The effect of CCG on thromboelastogram (TEG) of mice whole blood, CCG was at 50 µg/ml. Data were presented as mean ± SD (n = 3).

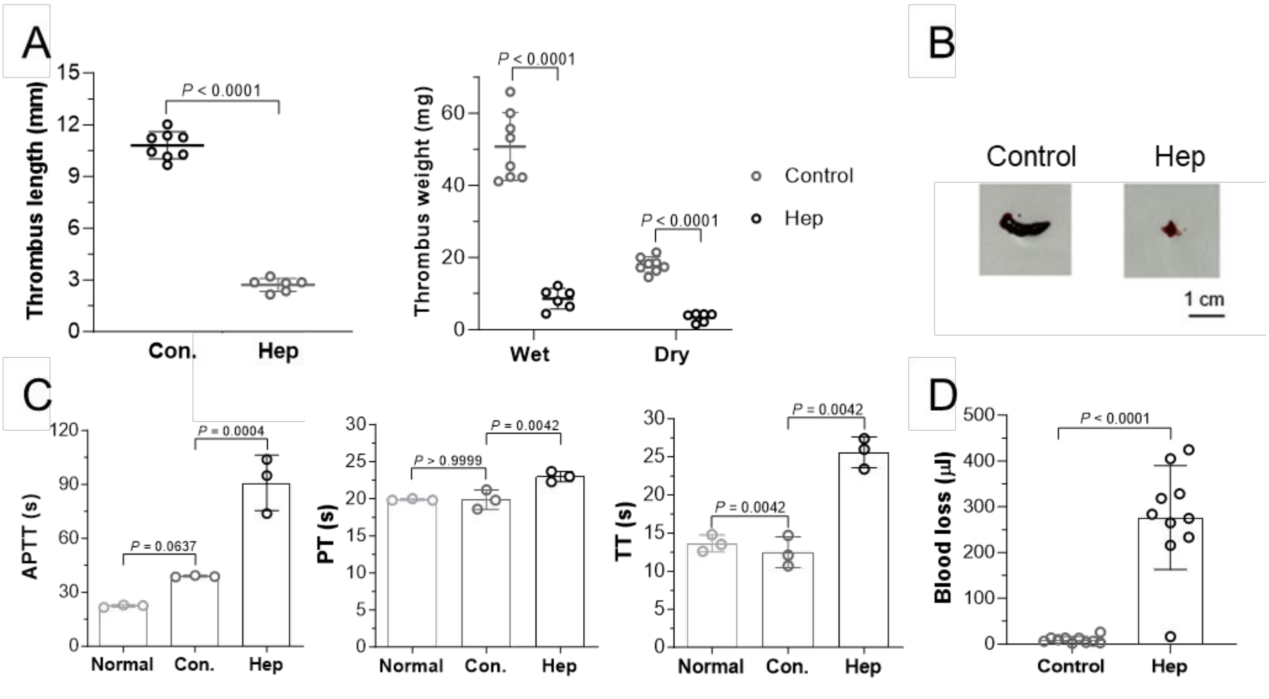

Fig. S12 The effect of heparin on thrombosis, plasma coagulation function and bleeding. (A) The effect of heparin (0.4 mg/kg) on thrombus length and thrombus weight in rat IVC thrombosis model (mean ± SD, n = 6–8).

(B) The representative thrombus pictures from rat IVC thrombosis model. (C) Effect of heparin on the coagulation function of rat plasma (mean ± SD, n = 3). (D) The effect of heparin (2.8 mg/kg) on mouse tail bleeding (mean ± SD, n = 10).

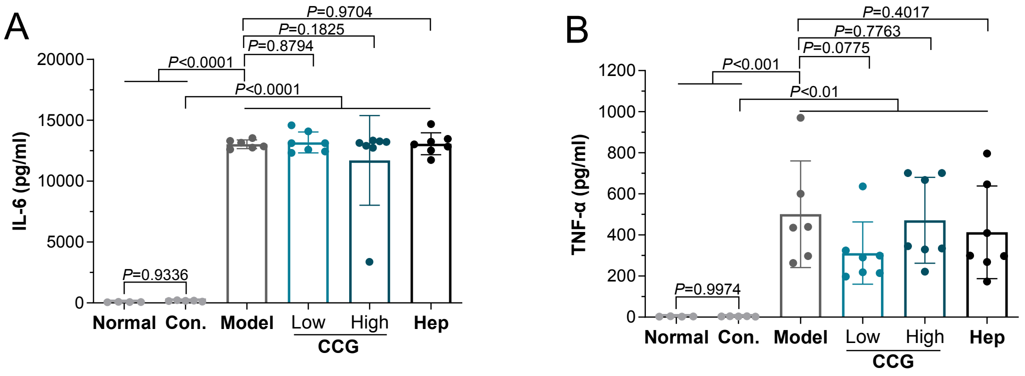

Fig. S13 The inflammation factor levels of plasma from endoxemia mice. (A–B) The plasma IL-6 (A) and TNF-α (B) levels of mice. Data were presented as mean ± SD (n = 6–7, except that n=4 and n=5 for normal and control group, respectively). Normal group are healthy mice, control group subjected to IVC ligation, all the other groups subjected to IVC ligation and LPS injection with or without compound treatment.

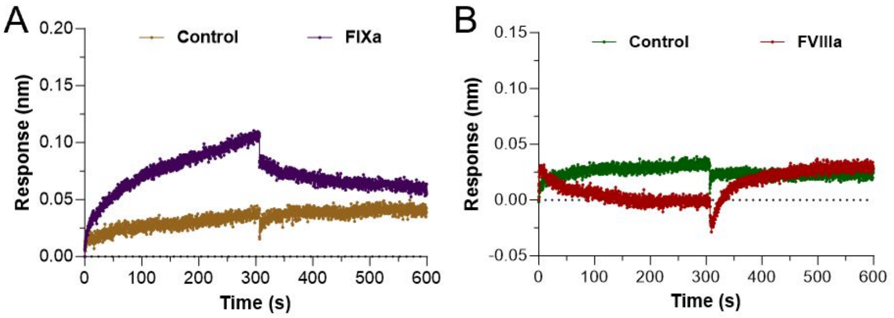

Fig. S14 The binding of FIXa (A) and FVIIIa (B) to CCG. CCG was biotinylated, and immobilized to streptavidin sensor, then CCG was interacted 40 nM FIXa and 40 nM FVIIIa, the binding signal of FIXa-CCG or FVIIIa-CCG were detected.

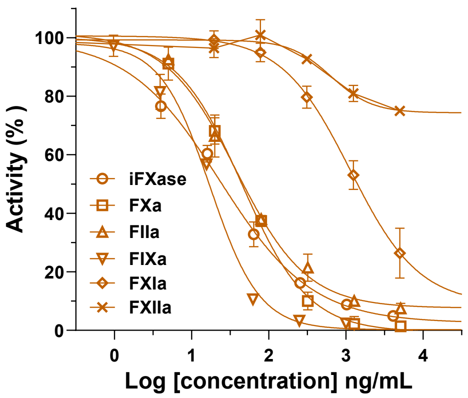

Fig. S15 Effect of enoxaparin on the activity of different coagulation factors. The effect of enoxaparin on iFXase was detected without AT, and its effects on FXa, FIIa, FIXa, FXIa and FXIIa were detected in the presence of AT. Data were presented as mean ± SD, experiments were conducted in duplicate.

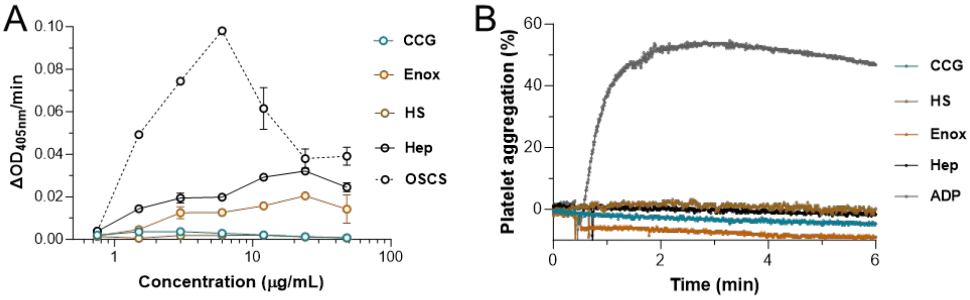

Fig. S16 Effect of GAGs on plasma contact activation (A) and platelet aggregation (B). Data were presented as mean ± SD (A) or representative image (B), experiments were conducted in duplicate.

Table S1. The retention time and molecular weight of CCG, CCG-MC and dCCG [a]

Compd.	RT (min)	Mp (kDa)	Mn (kDa)	Mw (kDa)	Mz (kDa)	PD
CCG	15.5	24.3	13.8	32.6	97.7	2.3
CCG-MC	16.0	16.6	10.5	19.2	36.7	1.8
dCCG	17.2	6.8	5.3	7.0	8.9	1.3

[a] Mp: peak molecular weight; Mn: number-average molecular weight; Mw: weight-average molecular weight; Mz (kDa): Z-average molecular weight; PD: polydispersity index.

Table S2. ^1H NMR and ^{13}C NMR data of sugar residues in dCCG [a]

Sugar residues	Position	^1H (δ ppm)	^{13}C (δ ppm)
A: 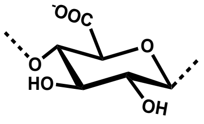	1	4.53	105.3
	2	3.42	75.9
	3	3.76	78.2
	4	3.81	80.9
	5	3.91	80.0
	6	-	177.4
B: 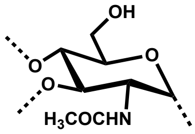	1	5.04/ 5.16	97.2/ 99.3
	2	4.26/ 4.21	56.2/ 55.9
	3	3.94/ 4.04	75.8/ 75.8
	4	3.70/ 3.75	77.0/ 77.0
	5	3.88/ 3.88	74.4/ 74.4
	6	3.88/ 3.88	62.3/ 62.3
	7	-	177.4/ 177.4
	8	2.07/ 2.07	25.3/ 25.3
C: 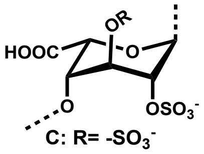 C: R= $-SO_3^-$ C': R= -H	1	5.28/ 5.06	101.4/ 103.3
	2	4.55/ 4.53	74.1/ 79.9
	3	4.78/ 4.23	71.8/ 69.2
	4	4.22/ 3.75	71.6/ 78.1
	5	4.88/ 5.01	70.1/ 70.8
	6	-	178.0/ 178.0
D: 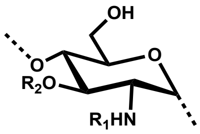 D: R_1= $-SO_3^-$; R_2= -H D': R_1= R_2= $-SO_3^-$ D'': R_1= $-COCH_3$; R_2= -H	1	5.46/ 5.40/ 5.09	99.9/ 101.8/ 97.1
	2	3.12/ 3.18/ 4.01	57.8/ 57.8/ 55.8
	3	3.90/ 4.59/ 3.88	74.4/ 74.0/ 74.0
	4	3.75/ 3.95/ 3.72	78.8/ 74.3/ 82.3
	5	3.85/ 3.85/ 3.85	72.6/ 72.6/ 72.6
	6	3.88/ 3.88/ 3.88	61.9/ 61.9/ 61.9
	7	-	177.4/ 177.4/ 177.4
	8	2.11/ 2.11/ 2.11	25.2/ 25.2/ 25.2
E: 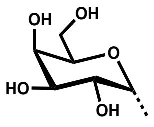	1	5.12	101.8
	2	3.69	70.8
	3	3.92	71.7
	4	4.05	72.1
	5	4.72	72.6

[a] Detected in D_2O.

Table S3. 2D NMR correlation data of monosaccharides within CCG [a]

COSY	TOCSY	ROESY	HMBC	HSQC-TOCSY
A: →4-D-GlcA-1β→				
H1/H2; H2/H3	H1/H2; H1/H3; H1/H4; H2/H3; H2/H4;	H2/H3; H2/H4; H3/H1;	H2/C1; H2/C3; H3/C1; H3/C2; H3/C4; H4/C3; H4/C5; H4/C6; H5/C1;	H2/C1; H2/C3; H2/C4; H2/C5; H3/C1; H3/C2; H3/C5; H4/C5;
B: →4-D-GlcNAc-1α→				
H1/H2; H1′/H2′; H2/H3; H3′/H2′; H3/H4; H4/H5;	H1/H2; H1/H3; H1′/H3′; H1/H5; H2/H3; H2/H4; H2/H5;	H2/H1; H2/H3; H2′/H3′; H2′/H4′; H3′/H5′; H4/H2; H4′/H2′; H6/H2;	H1/C3; H2/C5; H2/C3; H2/C7; H3/C4; H8/C7;	H3/C2; H5/C2;
C: →4-L-IdoA2S-1α→ or →4-L-IdoA2S3S-1α→				
H1′/H2′; H3/H4; H3′/H4′; H5/H4;	H1′/H2′; H2/H1; H2/H4; H3/H1; H3/H2; H3/H4; H4/H1; H4/H5; H5′/H1′;	H1/H2; H4′/H3′; H4/H5;	H1/C2; H1/C3; H1/C5; H2/C3; H2/C4; H3/C1; H3/C2; H3/C5; H4/C2; H4/C3; H4/C6; H4′/C6′; H5/C1; H5/C3; H5/C6; H5′/C6′;	H1′/C2′; H2′/C1′; H2/C3; H2′/C3′; H2′/C4′;
D: →4-D-GlcN2S-1α→, →4-D-GlcN2S3S-1α→ or →4-D-GlcNAc-1α→				
H1/H2; H1′/H2′; H1″/H2″; H2/H3; H3′/H2′; H3″/H2″; H3′/H4′;	H1/H2; H1/H3; H1″/H2″; H2/H3; H2/H5; H3/H4; H3′/H4′; H3′/H6′; H4/H2; H5′/H1′;	H2″/H4″	H1/C3; H3/C2; H3/C4; H4′/C2′; H4/C3; H4′/C6′; H8/C7;	H2/C3; H3/C2; H3″/C4″; H4/C5;
E: D-Gal-1α→				
H1/H2; H3/H4; H5/H6;	H1/H2; H1/H3; H2/H3; H2/H4; H4/H1; H4/H3; H5/H2; H5/H3; H1/H2;	H1/H2; H2/H3; H3/H5; H4/H5; H6/H5;	H1/C2; H1/C3; H1/C5; H4/C2; H4/C3; H5/C6; H6/C4; H6/C5;	H1/C3; H2/C4; H5/C6; H6/C5;

[a] The numbers indicated the positions of ^{1}H and ^{13}C in the sugar residues; detected in D_2O.

Table S4. 2D NMR correlation data of glycosidic bonds within CCG[a]

HMBC	ROESY
H_{B4}/C_{A1}; H_{B3}/C_{E1}; H_{E1}/C_{B3}; H_{C4}/C_{B1}; H_{C4}/C_{B1}; H_{C1}/C_{D4}; H_{D4}/C_{C1}; H_{A4}/C_{D1}; $H_{D''4}/C_{C'1}$	H_{B4}/H_{A1}; H_{B3}/H_{E1}; H_{C4}/H_{B1}; H_{C4}/H_{B1}; H_{C1}/H_{D4}; H_{D4}/H_{C1}; H_{D1}/H_{A4}; H_{A4}/H_{D1}; H_{A2}/H_{E5}; H_{E5}/H_{A1}; $H_{C'1}/H_{D'4}$; $H_{D'4}/H_{C2}$; $H_{A4}/H_{D''1}$; $H_{D4}/H_{C'1}$

[a] The numbers indicated the positions of ^{1}H and ^{13}C in the sugar residues; detected in D_2O.

Table S5. Effects of CCG on plasma coagulation time and coagulation factor

Compd.	Mw (kDa)	$EC_{2.0\times}$ (μg/ml)			IC_{50} (ng/ml)					
		APTT	PT	TT	FIIa/AT	FXa/AT	iFXase	FIXa/AT	FXIa/AT	FXIIa/AT
CCG	32.6	95.7	>200	>160	> 5000	> 5000	25.8	> 5000	> 250	> 5000
CCG-MC	19.2	>128	>200	>160	> 5000	> 5000	> 4000	-	-	-
AFG	697	>128	>200	>160	>1000	> 5000	> 4000	-	-	-
HLG	347	>128	>200	>160	> 5000	> 5000	> 4000	-	-	-
HS	37	53.4	>200	39.4	683.3	385.7	> 1000	-	-	-
Enox	4.5	6.6	>200	2.6	52.6	44.6	94.1	17.3	1142	> 5000
Hep	18	0.9	140.1	0.4	10.7	21.3	13.6	18.8	65.3	> 5000

Table S6. Effects of GAGs on thrombosis in rat DVT model

Treatment	Dose (mg/kg)	Thrombus length		Wet weight		Dry weight	
		(mm)	inhibition ratio (%)	(mg)	inhibition ratio (%)	(mg)	inhibition ratio (%)
Model	-	10.8 ± 0.8	/	50.7 ± 9.4	/	18.0 ± 2.1	/
	5	8.5 ± 0.6	21.0	35.8 ± 4.7	29.5	11.8 ± 2.2	34.1
CCG	10	4.7 ± 2.2	56.2	16.3 ± 9.6	67.9	5.5 ± 1.7	69.6
	20	1.6 ± 0.4	84.7	1.0 ± 0.7	98.1	0.3 ± 0.2	98.1
HS	1.25	4.2 ± 1.4	61.5	16.8 ± 9.5	66.8	5.9 ± 3.1	67.4
Enox	0.8	3.9 ± 1.7	63.8	13.7 ± 4.9	73.1	4.7 ± 1.9	73.8
Hep	0.4	2.7 ± 0.4	74.9	8.6 ± 2.9	83.1	3.4 ± 1.3	80.9

Table S7. Effects of GAGs on thrombosis in rat A-V shunt thrombosis model

Treatment	Dose (mg/kg)	Wet weight		Dry weight	
		(mg)	inhibition ratio (%)	(mg)	inhibition ratio (%)
Model	-	50.3 ± 3.9	/	11.5 ± 2.1	/
CCG	10	34.7 ± 6.8	30.9	8.6 ± 2.2	25.4
HS	5	17.9 ± 6.7	64.4	4.5 ± 1.8	60.7
Enox	5	18.0 ± 8.8	64.3	4.9 ± 2.5	57.5

Table S8. Effects of GAGs on thrombosis in mouse endoxemia-enhanced DVT model

Treatment	Dose (mg/kg)	Thrombosis incidence	Thrombus length		Wet weight		Dry weight	
			(mm)	inhibition ratio (%)	(mg)	inhibition ratio (%)	(mg)	inhibition ratio (%)
Control	-	3/8	2.3 ± 1.2	/	3.3± 2.9	/	1.3 ± 0.5	/
Model	-	10/15	3.5 ± 1.2	/	3.1 ± 2.2	/	1.3 ± 0.8	/
CCG	5	6/12	1.7 ± 0.1	50.4	1.2 ± 1.0	60.7	0.5 ± 0.5	59.2
	10	1/11	1.5 ± 0.0	57.3	0.3 ± 0.0	91.6	0.3 ± 0.0	81.1
Hep	1	2/9	1.8 ± 1.2	47.7	0.8 ± 0.8	72.9	0.4 ± 0.3	71.6

Table S9. The kinetic parameters of protein interaction with GAG

Ligand	Analyte	k_{on} ($M^{-1}\cdot s^{-1}$)	k_{dis} (s^{-1})	K_D (M)	Full R^2
CCG	FIXa	1.2×10^4 ($\pm$ 9.5 $\times$ 10)	5.6×10^{-4} ($\pm 2.9 \times 10^{-6}$)	4.6×10^{-8} ($\pm 4.4 \times 10^{-10}$)	0.992
	AT	-[a]	-[a]	-[a]	-[a]
Hep	FIXa	2.9×10^4 ($\pm 1.5 \times 10^2$)	5.2×10^{-4} ($\pm 2.6 \times 10^{-6}$)	1.8×10^{-8} ($\pm 1.3 \times 10^{-10}$)	0.993
	AT	6.2×10^4 ($\pm 1.7 \times 10^3$)	4.9×10^{-3} ($\pm 3.4 \times 10^{-5}$)	8.8×10^{-8} ($\pm 2.3 \times 10^{-9}$)	0.954

[a] The binding signals between CCG and AT were undetectable.

Table S10. Structural comparison of polysaccharides from different snail species

Polysaccharide	Structure	Snail species	Reference
Snail GAG		*Achatina fulica*	Kim YS, *et al. J Biol Chem* 1996; **271**: 11750-11755
AF-GAG	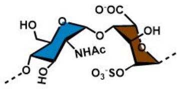	*Achatina fulica*	Liu J, *et al. Carbohydr Polym* 2018; **181**: 433-441
AFG		*A. fulica* "white"	Wu Y, *et al. Carbohydr Polym* 2020; **247**: 116682
HLG	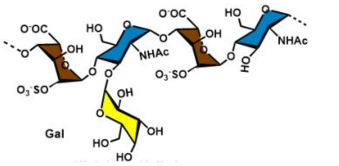	*Helix lucorum*	Li Y, *et al. Carbohydr Polym* 2025; **348**: 122900
CCG	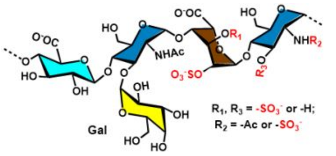	*Camaena cicatricosa*	This work
